# The effects of gross motor exercises on perception and praxis in children with motor coordination difficulties–randomized controlled study

**DOI:** 10.3389/fneur.2026.1891034

**Published:** 2026-09-07

**Authors:** Bartosz Bagrowski, Bartosz Grobelny, Andrew Dalziell, Paweł Górny, Ewa Mojs

**Affiliations:** 1 Department of Clinical Psychology, Doctoral School, Poznan University of Medical Sciences, Poznan, Poland; 2 Department of Clinical Psychology, Poznan University of Medical Sciences, Poznan, Poland; 3 Moray House School of Education and Sport, University of Edinburgh, Edinburgh, United Kingdom; 4 Non-public Psychological and Pedagogical Counseling Center “Otwarte Drzwi”, Poznan, Poland

**Keywords:** bilateral coordination, developmental coordination disorder, dyspraxia, neurodevelopement, neuropsychology, physiotherapy, praxis, somatognosia

## Abstract

**Introduction:**

Child psychomotor development is holistic, and pediatric rehabilitation increasingly emphasizes multidisciplinary approaches. Movement interventions can influence not only physical but also cognitive and executive functions. Midline crossing exercises may stimulate frontal–parietal networks, including the dorsolateral prefrontal cortex and superior parietal cortex, involved in praxis and executive control. However, their long-term effects in children with Developmental Coordination Disorder (DCD) remain unclear.

**Methods:**

This study examined whether midline crossing exercises improve perceptual and praxis functions more than exercises without this element, and explored their relationship with bilateral integration. Twenty-two children aged 6–9 with DCD symptoms participated in a randomized trial. They were assigned to a research group (with midline crossing) or a control group (without it), both following a Bilateral Integration program. The intervention lasted 9–10 weeks within a 15–16-week cycle, with assessments of perception, praxis, and bilateral integration processes at three time points (t_0_, t_1_, t_2_). Statistical analyses primarily included ANOVA tests and correlation analysis.

**Results:**

Both groups showed significant improvements in Sequencing Praxis and Bilateral Motor Coordination, with no between-group differences at specific time points. However, improvements appeared earlier in the research group (t_1_), while the control group improved only at follow-up (t_2_). Additional gains in specific praxis tasks, lower limb coordination, and task speed were observed only in the research group. Perceptual changes were minimal overall.

**Discussion:**

Bilateral Integration exercises improve praxis, coordination, and task performance speed in children with DCD symptoms. Midline crossing may accelerate therapeutic effects and selectively enhance certain functions, supporting integrated models of development.

## Graphical abstract

**Figure:**
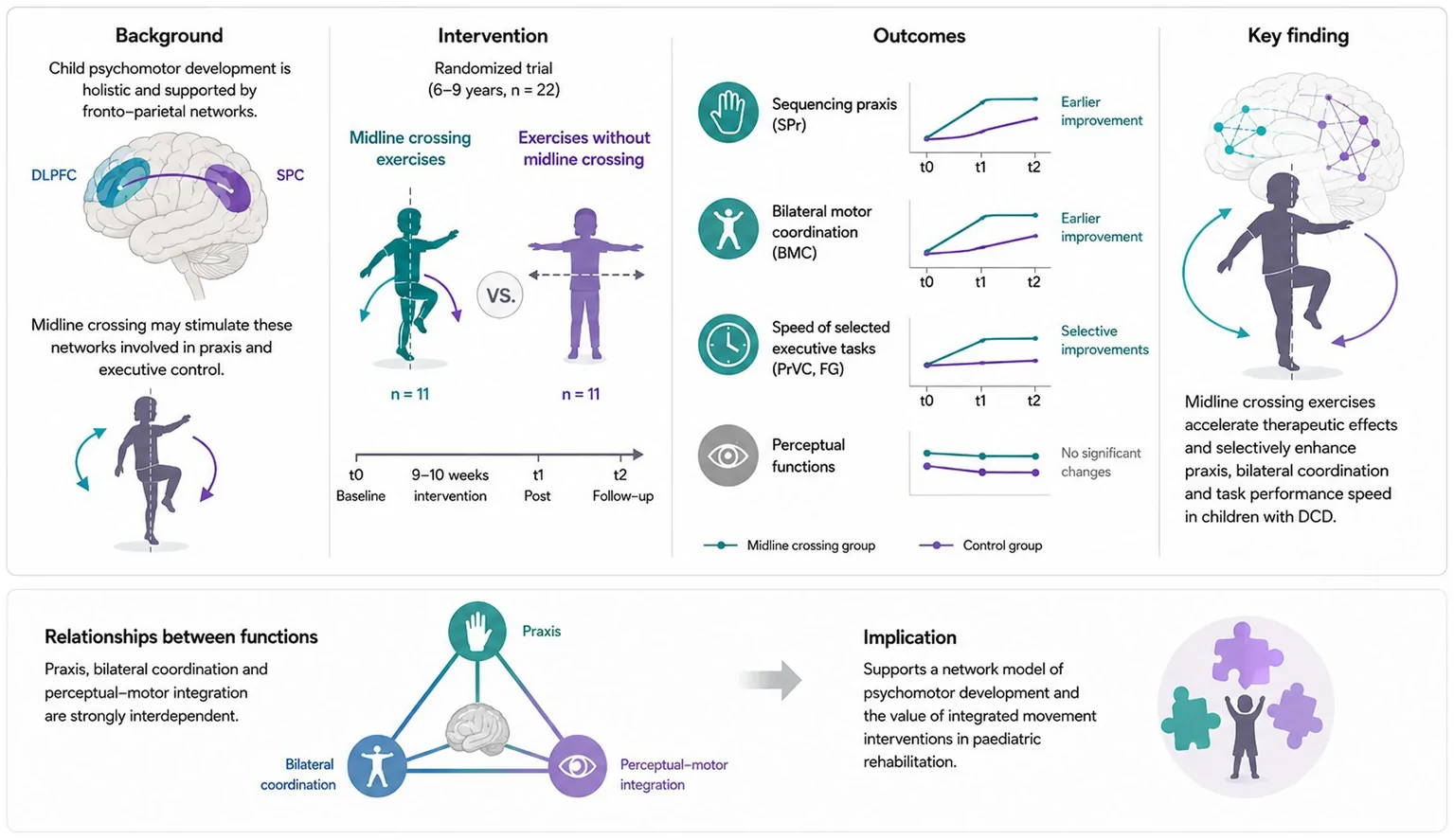

## Introduction

Children’s psychomotor development is holistic, and therapeutic interventions used during developmental age often intersect various disciplines (e.g., physiotherapy, psychology, and pedagogy). This is why multidisciplinary approaches to pediatric rehabilitation and child development support are increasingly emphasized (1–3). One issue that demonstrates this multifaceted and multidirectional approach is the impact of physical interventions on mental functioning. Numerous researchers emphasize the importance of movement interventions for mental health and overall well-being (4), as well as for neuromuscular coordination and mental activity (5, 6). Other studies have shown that focusing attention and awareness on a motor task positively influences learning mechanisms, autonomic regulation, and body schema development, as well as increases neuroplasticity (7, 8). In developmental age, coordination exercises using the Bilateral Integration method devised by Sheila Dobie OBE or based on its assumptions were associated with improved somatognosia, balance, postural control, motor coordination, interhemispheric cooperation, motor learning, movement planning, academic skills, visual–spatial processing, working memory, and verbal reasoning (9–11). Complex motor planning and spatial integration are coordinated by the prefrontal and parietal cortices. In this context it is also necessary to mention that electroencephalographic studies using the PNF (Proprioceptive Neuromuscular Facilitation) method have shown that functional movements that cross the midline of the body are associated with additional activation of the dorsolateral prefrontal cortex (DLPFC) and superior parietal cortex (SPC) – cortical areas responsible for some cognitive and executive functions (12). These movements are also associated with the excitation of signals that are treated as correlates of plasticity (13). However, long-term changes associated with such exercises have not been studied so far.

The main aim of this study was to verify whether exercises using functional movements that cross the midline would improve the cognitive and executive functions that the DLPFC and SPC are also responsible for. For this purpose, Bilateral Integration by Sheila Dobie OBE, a method with proven positive effects on the psychomotor development of children and adolescents (9–11), was used. This effect was examined in a group of children with symptoms of Developmental Coordination Disorder (DCD), in whom one of the main symptom clusters is impaired motor control and coordination skills. In the context of DCD symptoms, attention is drawn to dysfunction (but not damage) in the prefrontal region (of which the DLPFC is a part), the parietal region (of which the SPC is a part), and the cerebellum (14). Identifying an intervention program that would specifically support the cortical areas that are dysfunctional in children with DCD symptoms would therefore be very valuable and could, to some extent, align with the trend of personalized medicine.

This article is the most important part of the publication series “Assessment of the impact of motor exercises according to the Bilateral Integration program (by Sheila Dobie OBE) on cognitive functions among early school children with symptoms of Developmental Coordination Disorder (DCD).” A detailed theoretical framework is described in the initial review article (15). The Study Protocol article describes in detail all the key assumptions of the project (16), and the Preliminary Report demonstrates that midline crossing exercises were associated with faster functional improvement in Sequencing Praxis and Bilateral Motor Coordination (17). The main justification for this work is the detailed discussion of all parameters examined in the project (which have not been analyzed previously), not just the main results (which were analyzed in the Preliminary Report).

In this study, the functional indicators examined regarding cognitive and executive functioning will include mainly perceptual processes and praxis. The main research questions – already posed in the Study Protocol – are:

1 Do exercises with crossing the body’s midline improve perceptual processes more than exercises without crossing the body’s midline?
2 Do exercises with crossing the body’s midline improve praxis processes more than exercises without crossing the body’s midline?
3 Are perceptual and praxis processes clearly related to indicators of bilateral integration processes?

## Materials and methods

Initially, 32 participants were enrolled in the project, but for various reasons, the full diagnostic and therapeutic cycle was completed with 22 participants (early school-age children with DCD symptoms). A detailed flowchart and sample size analysis are included in the Preliminary Report (17). Although similar studies estimated a minimum sample size of 27 participants, analyses of the parameters examined in the Preliminary Report showed that the power of the statistical tests was sufficiently high that some high effect sizes would have been clearly visible with just four participants per group (17). Participants were eligible if they were between 6 and 9 years of age and presented symptoms consistent with dyspraxia in the absence of organic impairment (even without formal diagnosis of DCD). Exclusion criteria comprised the lack of written informed consent from a parents/guardians, as well as a documented intellectual disability severe enough to prevent comprehension of the assessment procedures or intervention tasks. The study was approved by the Bioethics Committee of the Poznan University of Medical Sciences – Resolution No. 847/22 of November 03, 2022 with later changes accepted by Resolution No. 214/24 of March 07, 2024.

Twenty two participants were randomly allocated to either the intervention or control group immediately prior to baseline functional assessment. Group assignment was based on a simple allocation sequence generated according to the order of enrolment. Participants assigned an odd identification number were allocated to the control group, whereas those assigned an even number were allocated to the intervention group. The allocation sequence was implemented without prior review of questionnaire data. The main principle for assigning numbers was the order in which the participant registered for the study - identification numbers were assigned consecutively at enrolment and were not reassigned in the event of participant withdrawal. Participants were blinded to their group, and the interventionist was not blinded, as he or she needed to know which exercise type to assign to each participant. However, to ensure the results were not biased, all tests were analyzed only after each participant had completed the full diagnostic and training cycle.

Each participant was to participate in a 15–16 week study cycle, which consisted of a 9–10 week diagnostic and therapeutic cycle and a 6–7 week break before the follow–up assessment. Initially, the entire cycle was planned to last 10 weeks (6 weeks of intervention and 4 weeks of rest). However, due to participant illnesses or absences due to other factors, the entire cycle was extended. Details regarding the actual duration of the diagnostic and therapeutic cycle, the frequency of exercises, and the duration of the exercise session can be found in the Preliminary Report (17).

The two groups differed with respect to the intervention protocol. In both conditions, exercises were derived from the Bilateral Integration program. In the Control group, activities were performed without incorporating movements across the body midline (Body Lifts, Basic Angels, Alternating Exercise). In contrast, the Research group completed tasks that predominantly required crossing the midline (Body Lifts, Cross Overs, Sun–Wind–Rainbow). A comprehensive description of the exercise protocols is provided in the Study Protocol (16). The groups did not differ significantly in terms of age, body weight, BMI, comorbidities, neurodevelopmental questionnaire scores, sensorimotor development questionnaire scores, biomechanical parameters, static and dynamic balance levels, and physical activity levels. A detailed analysis in this regard was presented in the Preliminary Report (17). Statistically significant (although slightly) differences were observed between the groups in terms of height and the degree of shin torsion, however considered independently, these parameters appear to have limited relevance. No evidence was identified indicating an association between tibial torsion and cognitive, executive, or praxis-related processes; therefore, this difference was not regarded as clinically meaningful. With respect to body height, available findings suggest no association between stature and mental health conditions (18). Although the referenced study pertains specifically to intelligence (i.e., the intellectual domain), additional research likewise indicates no established relationship between height and broader cognitive functioning (19).

The main functional tests were performed at three time points: before the intervention (t_0_), after the 6–week intervention (t_1_), and after a 4–week break after the intervention (t_2_). The tests used included: Kinaesthesia (KIN), Localization of Tactile Stimuli (LTS), Manual Form Perception (MFP), Figure–Ground Perception (FG), Postural Praxis (PPr), Sequencing Praxis (SPr), Constructional Praxis (CPr), Praxis on Verbal Command (PrVC), Bilateral Motor Coordination (BMC) and Gross Motor Coordination (GMC) (9, 20–23).

Statistical analyses were conducted in accordance with established methodological guidelines (24, 25) using PQStat software (version 1.8.6.122). Statistical significance was set at *p* < 0.05. The distribution of all variables was initially assessed with the Shapiro–Wilk test. Subsequent analyses were selected based on distribution characteristics and variance assumptions. Homogeneity of variance was evaluated using Levene’s test. Intergroup and intragroup effects for the primary functional outcomes measured at three time points were analyzed using ANOVA for independent samples and ANOVA for dependent samples, as appropriate. When assumptions of normality were met, parametric ANOVA models were applied, with Welch’s correction used where homogeneity of variance was violated. If the sphericity assumption was not satisfied (as indicated by Mauchly’s test), Greenhouse–Geisser correction was implemented. For non-normally distributed data, nonparametric alternatives were employed (Kruskal–Wallis one-way ANOVA by ranks for independent samples and the Friedman test for repeated measures). *Post hoc* comparisons were conducted using Tukey’s HSD test for parametric ANOVA or the Dunn test with Benjamini–Hochberg correction for nonparametric analyses to assess differences between groups and across the three measurement time points. To answer the third research question, correlation analysis was also necessary – the Pearson correlation coefficient or Spearman’s rank correlation coefficient was used to measure the correlation between variables depending on the parametric distribution of the variables.

## Results

Functional assessments were conducted in both groups at three measurement time points. The outcomes of the perception tests, along with the corresponding intergroup and intragroup comparisons, are presented in Table 1. Results of the praxis assessments and related between- and within-group analyses are summarized in Table 2. Findings from the bilateral integration tests, including intergroup and intragroup comparisons, are provided in Table 3.

**Table 1:** Results of the perception tests (and sub-tests) at three time points.

Measure	Group	Pre-test (t_0_)	Post-test (t_1_)	Follow-up testing (t_2_)	*p*-value ANOVA within group (effect on time)	Significant differences within group (post-hoc test)
KIN (Total)	Control	41.91 ± 19.29 36.4 [30.55–45.6]	43.15 ± 21.05 35.8 [31.7–42.35]	35.13 ± 9.13 38 [28.75–40.85]	*p = 0.178*	
	Research	40.01 ± 14.28 37.3 [30.1–51.25]	38.69 ± 9.08 38.1 [34.35–44.15]	43.96 ± 19.10 38.7 [35–46.55]	*p = 0.529*	
	*p*-value ANOVA between groups	*p = 0.974*	*p = 0.232*	*p = 0.507*		
KIN (Right)	Control	16.87 ± 6.11 15.6 [14.05–20.9]	21.55 ± 10.92 17.5 [15.35–23.15]	17.46 ± 5.63 19.2 [13.9–20.4]	*p = 0.761*	
	Research	19.07 ± 7.89 17.2 [14.6–22.15]	20.02 ± 4.95 19.6 [17.3–21.75]	22.53 ± 18.02 17.2 [14.65–22]	*p = 0.336*	
	*p*-value ANOVA between groups	*p* = 0.974	*p = 0.232*	*p = 0.507*		
KIN (Left)	Control	25.05 ± 16.56 20.8 [17.25–24.2]	21.6 ± 10.60 17.5 [15.9–22.8]	17.66 ± 5.09 18.6 [15.1–21.6]	*p = 0.320*	
	Research	20.94 ± 7.48 20.8 [15.85–26.8]	18.67 ± 7.16 17.5 [13.1–22.4]	21.44 ± 6.46 21.4 [17.8–23.7]	*p* = 0.626	
	*p*-value ANOVA between groups	*p = 0.974*	*p = 0.232*	*p* = 0.507		
LTS (Total)	Control	16.23 ± 5.46 14.7 [12.3–19.3]	13.88 ± 3.63 12.8 [10.7–16.9]	11.90 ± 3.00 10.2 [9.6–15.1]	*p = 0.148*	
	Research	13.25 ± 4.09 14.9 [9.85–16.35]	12.89 ± 4.28 12.2 [9.8–15.1]	14.29 ± 5.93 14 [10–14.75]	*p* = 0.722	
	*p*-value ANOVA between groups	*p* = 0.163	*p = 0.563*	*p = 0.269*		
LTS (Right)	Control	9.27 ± 4.32 8 [6.1–12.15]	6.82 ± 2.44 6 [5.25–8.1]	5.64 ± 1.97 5.2 [3.7–7.5]	*p = 0.148*	
	Research	6.49 ± 2.37 6.5 [5.65–8.1]	6.53 ± 2.42 5.5 [4.85–7.65]	8 ± 4.85 5.9 [4.55–9.4]	*p = 0.811*	
	*p*-value ANOVA between groups	*p* = 0.076	*p* = 0.782	*p = 0.250*		
LTS (Left)	Control	6.95 ± 2.16 7.1 [5.15–8.5]	7.06 ± 1.92 6.5 [5.95–7.65]	6.26 ± 1.33 5.8 [5.05–7.1]	*p* = 0.452	
	Research	6.75 ± 2.36 6.6 [5.55–8.55]	6.36 ± 2.62 5.2 [4.55–6.9]	6.29 ± 2.19 5.3 [4.75–8.1]	*p = 0.977*	
	*p*-value ANOVA between groups	*p* = 0.838	*p = 0.278*	*p* = 0.972		
MFP (Total)	Control	8.36 ± 1.29 8 [8–9.5]	7.91 ± 1.30 8 [7–9]	8.00 ± 1.26 8 [7–9]	*p* = 0.605	
	Research	7.91 ± 1.22 8 [7–9]	8.09 ± 1.38 8 [7–9]	8.18 ± 2.18 9 [7–10]	*p = 0.815*	
	*p*-value ANOVA between groups	*p* = 0.405	*p* = 0.753	*p = 0.064*		
MFP (Right)	Control	4 [3.5–5]	4 [3–4.5]	4 [3–4]	*p = 0.459*	
	Research	3 [3–4]	4 [4–5]	5 [4–5]	*p = 0.075*	
	*p*-value ANOVA between groups	*p = 0.142*	*p = 0.266*	*p = 0.178*		
MFP (Left)	Control	4 [4–5]	4 [3–5]	4 [4–5]	*p = 0.717*	
	Research	4 [4–5]	4 [3–4.5]	4 [3–5]	*p = 0.325*	
	*p*-value ANOVA between groups	*p = 0.749*	*p = 0.482*	*p = 0.859*		
MFP (Time) (Total)	Control	63.09 ± 24.77 53 [51–73]	60 ± 14.78 59 [50–72]	65.27 ± 29.59 57 [48–65.5]	*p = 0.695*	
	Research	83.45 ± 55.85 54 [51–103.5]	80.64 ± 34.48 83 [53–96]	69.45 ± 36.49 51 [47–85]	*p = 0.513*	
	*p*-value ANOVA between groups	*p = 0.767*	*p* = 0.083	*p = 0.742*		
MFP (Time) (Right)	Control	33 ± 11.09 31 [26.5–37.5]	33.36 ± 7.94 35 [28–37]	36 ± 17.01 33 [26.5–38]	*p = 0.909*	
	Research	43.73 ± 27.09 32 [26.5–48.5]	48.64 ± 22.57 47 [31.5–56]	42.09 ± 27.01 30 [24.5–51]	*p = 0.850*	
	*p*-value ANOVA between groups	*p = 0.554*	*p* = 0.055*	*p = 0.921*		
MFP (Time) (Left)	Control	26 [21.5–32.5]	29 [18–34.5]	24 [22.5–30]	*p = 0.761*	
	Research	23 [21.5–50.5]	27 [20.5–36]	23 [20.5–30.5]	*p = 0.303*	
	*p*-value ANOVA between groups	*p = 1.000*	*p = 0.645*	*p = 0.510*		
FG	Control	16.27 ± 2.24	16.73 ± 2.49	16.18 ± 3.22	*p* = 0.821	
	Research	15.36 ± 3.41	17.27 ± 2.94	16.82 ± 3.34	*p* = 0.219	
	*p*-value ANOVA between groups	*p* = 0.469	*p* = 0.644	*p* = 0.654		
FG (Errors)	Control	9 [8–10]	8 [7.5–8]	8 [8–10]	*p = 0.423*	
	Research	8 [7.5–10]	8 [8–9]	8 [8–8]	*p = 0.497*	
	*p*-value ANOVA between groups	*p = 0.710*	*p = 0.460*	*p = 0.337*		
FG (Time)	Control	153 ± 56.19 168 [101–185]	143.73 ± 30.50 144 [129–158]	113.55 ± 32.97 117 [97.5–129.5]	***p* = 0.002** **(η**^**2**^ **= 0.457)**	t_2_ < t_0_ (*p* = 0.002) t_2_ < t_1_ (*p* = 0.019)
	Research	144.82 ± 52.75 128 [111–174.5]	131.82 ± 43.35 136 [91–170]	140 ± 46.05 152 [114.5–169]	*p = 0.529*	
	*p*-value ANOVA between groups	*p* = 0.728	*p = 0.768*	*p* = 0.137		

KIN – Kinesthesia; LTS – Localization of Tactile Stimuli; MFP – Manual Form Perception; FG – Figure-Ground perception. Results are presented as mean with standard deviation (M ± SD). If at least one result of a given functional test did not meet the criteria for a normal distribution (the mean and standard deviation are underlined), the results are also presented as median with lower and upper quartiles (Me [Q1-Q3]). For parametric ANOVA, means should be compared, while for nonparametric ANOVA, medians should be compared. *p*-values obtained from nonparametric ANOVA (Friedman’s ANOVA for dependent measures and Kruskal-Wallis ANOVA for independent measures) are in italics. *p*-values that were statistically significant are bolded (bolding was not used for *post-hoc* test results, as only those that were significant are indicated in the table). For statistically significant results, the effect size was additionally determined in the form of η^2^ in parametric ANOVA. *Values calculated with Welch’s correction due to unequal variances.

**Table 2:** Results of the praxis tests (and sub-tests) at three time points.

Measure	Group	Pre-Test (t_0_)	Post-Test (t_1_)	Follow-up testing (t_2_)	*p*-value ANOVA within group (effect on time)	Significant differences within group (*post-hoc* test)
PPr	Control	15.73 ± 4.15	15.82 ± 5.31	16.64 ± 5.03	*p* = 0.108	
	Research	14.45 ± 4.80	14.27 ± 5.12	15.73 ± 6.28	*p* = 0.338	
	*p*-value ANOVA between groups	*p* = 0.514	*p* = 0.495	*p* = 0.712		
CPr	Control	13.18 ± 2.32 13 [12–15]	13.36 ± 2.34 13 [13–15]	14.82 ± 1.08 15 [14–16]	*p = 0.126*	
	Research	12.64 ± 3.91 14 [13.5–14.5]	14.27 ± 2.57 15 [14.5 = 15.5]	13.73 ± 2.97 15 [13.5–15.5]	*p = 0.159*	
	*p*-value ANOVA between groups	*p = 0.920*	*p = 0.177*	*p = 0.541*		
PrVC	Control	18.73 ± 4.36 21 [17–22]	19.55 ± 4.80 21 [19–22]	20.18 ± 3.37 21 [19.5–22]	*p = 0.376*	
	Research	18.55 ± 4.03 20 [16–22]	19.09 ± 5.20 22 [15–22.5]	19.36 ± 5.73 23 [14.5–24]	*p = 0.567*	
	*p*-value ANOVA between groups	*p = 0.894*	*p = 0.617*	*p = 0.595*		
PrVC (Time)	Control	71 ± 15.01 70 [63–78]	73.91 ± 17.91 73 [61–84]	66.18 ± 18.20 69 [49.5–75.5]	*p* = 0.329	
	Research	73.55 ± 17.31 70 [63–78.5]	74.09 ± 22.58 64 [58.5–82]	63.55 ± 16.38 60 [55.5–63]	***p = 0.011*** ***(W = 0.408)***	t_2_ < t_0_ (*p* = 0.021) t_2_ < t_1_ (p = 0.021)
	*p*-value ANOVA between groups	*p* = 0.716	*p = 0.743*	*p = 0.622*		
SPr (Total)	Control	47.82 ± 18.95	51.09 ± 19.83	55.64 ± 22.20	***p* = 0.031** **(η**^**2**^ **= 0.293)**	t_2_ > t_0_ (*p* = 0.025)
	Research	38.55 ± 18.73	50.55 ± 20.32	55.00 ± 21.51	***p* < 0.001** **(η**^**2**^ **= 0.670)**	t_1_ > t_0_ (*p* < 0.001) t_2_ > t_0_ (*p* < 0.001)
	*p*-value ANOVA between groups	*p* = 0.262	*p* = 0.950	*p* = 0.946		
SPr (Hands)	Control	39.45 ± 12.75	41.45 ± 12.73	43.82 ± 13.51	*p* = 0.137	
	Research	34.45 ± 16.25	42.73 ± 15.61	43.73 ± 14.52	***p* < 0.001** **(η**^**2**^ **= 0.541)**	t_1_ > t_0_ (*p* = 0.002) t_2_ > t_0_ (*p* < 0.001)
	*p*-value ANOVA between groups	*p* = 0.431	*p* = 0.836	*p* = 0.988		
SPr (Fingers)	Control	8.36 ± 6.98	9.64 ± 8.39	11.82 ± 9.54	*p* = 0.146	
	Research	4.09 ± 3.91	7.81 ± 6.65	11.27 ± 8.20	***p* = 0.002** **(η**^**2**^ **= 0.455)**	t_2_ > t_0_ (p = 0.002)
	*p*-value ANOVA between groups	*p* = 0.092	*p* = 0.580	*p* = 0.887		

PPr – Postural Praxis; CPr – Constructional Praxis; PrVC – Praxis on Verbal Command; SPr – Sequencing Praxis. Results are presented as mean with standard deviation (M ± SD). If at least one result of a given functional test did not meet the criteria for a normal distribution (the mean and standard deviation are underlined), the results are also presented as median with lower and upper quartiles (Me [Q1-Q3]). For parametric ANOVA, means should be compared, while for nonparametric ANOVA, medians should be compared. *p*-values obtained from nonparametric ANOVA (Friedman’s ANOVA for dependent measures and Kruskal-Wallis ANOVA for independent measures) are in italics. *p*-values that were statistically significant are bolded (bolding was not used for post-hoc test results, as only those that were significant are indicated in the table). For statistically significant results, the effect size was additionally determined in the form of η^2^ in the case of parametric ANOVA and Kendall’s *W* in the case of non-parametric ANOVA.

**Table 3:** Results of the bilateral integration tests (and sub-tests) at three time points.

Measure	Group	Pre-test (t_0_)	Post-test (t_1_)	Follow-up testing (t_2_)	*p*-value ANOVA within group (effect on time)	Significant differences within group (*post-hoc* test)
BMC (Upper limbs)	Control	7.36 ± 4.48	8.09 ± 5.58	11 ± 4.80	***p* = 0.007** **(η**^**2**^ **= 0.389)**	t_2_ > t_0_ (*p* = 0.008) t_2_ > t_1_ (*p* = 0.035)
	Research	5.73 ± 4.82	9.09 ± 5.56	9.64 ± 6.61	**p = 0.008** **(η**^**2**^ **= 0.381)**	t_1_ > t_0_ (*p* = 0.029) t_2_ > t_0_ (*p* = 0.011)
	*p*-value ANOVA between groups	*p* = 0.419	*p* = 0.678	*p* = 0.586		
BMC (Lower limbs)	Control	2.18 ± 1.94	2.91 ± 1.97	2.73 ± 2.20	*p* = 0.355	
	Research	1.73 ± 1.68	3.00 ± 2.28	3.18 ± 2.68	***p* = 0.034** **(η**^**2**^ **= 0.288)**	t_2_ > t_0_ (*p* = 0.043)
	*p*-value ANOVA between groups	*p* = 0.563	*p* = 0.921	*p* = 0.668		
BMC (Total)	Control	8.60 ± 5.56	11.00 ± 7.09	13.73 ± 6.02	***p* = 0.013** **(η**^**2**^ **= 0.350)**	t_2_ > t_0_ (*p* = 0.009)
	Research	7.45 ± 6.42	12.09 ± 7.60	12.82 ± 9.15	*p* = 0.020** (η^**2**^ **= 0.387)**	t_1_ > t_0_ (*p* = 0.027) t_2_ > t_0_ (*p* = 0.010)
	*p*-value ANOVA between groups	*p* = 0.669	*p* = 0.731	*p* = 0.786		
GMC (Crawling)	Control	2.45 ± 1.13 3 [2–3]	2.09 ± 1.22 2 [1–3]	2.27 ± 1.19 3 [2–3]	*p = 0.756*	
	Research	2.18 ± 1.25 3 [1–3]	1.45 ± 1.13 1 [1–2]	2.09 ± 1.45 2 [1–3]	*p* = 0.150	
	*p*-value ANOVA between groups	*p = 0.621*	*p* = 0.219	*p = 0.781*		
GMC (Creeping)	Control	1 [0–1]	1 [1–1]	1 [0–1]	*p = 0.368*	
	Research	0 [0–1]	1 [0–1]	0 [0–1]	*p = 0.549*	
	*p*-value ANOVA between groups	*p = 0.443*	*p = 0.091*	*p = 0.403*		
GMC (March)	Control	0 [0–1]	0 [0–0]	0 [0–0.5]	*p = 0.549*	
	Research	0 [0–0]	0 [0–1]	0 [0–1]	*p = 0.692*	
	*p*-value ANOVA between groups	*p = 0.372*	*p = 0.311*	*p = 0.386*		
GMC (Skipping)	Control	2.45 ± 0.93 2 [2–3]	1.82 ± 1.33 1 [1–2.5]	1.82 ± 0.98 2 [1–2.5]	*p = 0.114*	
	Research	1.80 ± 0.92 1.5 [1–2.75]	1.82 ± 1.08 1 [1–2.5]	2.36 ± 1.29 3 [1.5–3]	*p = 0.338*	
	*p*-value ANOVA between groups	*p = 0.130*	*p = 0.916*	*p* = 0.277		
GMC (Total)	Control	6.09 ± 2.02	5.09 ± 1.81	4.91 ± 1.04	*p* = 0.108	
	Research	4.45 ± 1.69	4.27 ± 1.10	5.27 ± 2.00	*p* = 0.338	
	*p*-value ANOVA between groups	*p* = 0.053	*p* = 0.216	*p* = 0.601*		

BMC – Bilateral Motor Coordination test; GMC – Gross Motor Coordination test. Results are presented as mean with standard deviation (M ± SD). If at least one result of a given functional test did not meet the criteria for a normal distribution (the mean and standard deviation are underlined), the results are also presented as median with lower and upper quartiles (Me [Q1-Q3]). For parametric ANOVA, means should be compared, while for nonparametric ANOVA, medians should be compared. *p*-values obtained from nonparametric ANOVA (Friedman’s ANOVA for dependent measures and Kruskal-Wallis ANOVA for independent measures) are in italics. *p*-values that were statistically significant are bolded (bolding was not used for post-hoc test results, as only those that were significant are indicated in the table). For statistically significant results, the effect size was additionally determined in the form of η^2^ in parametric ANOVA. *Values calculated with Welch’s correction due to unequal variances. **Values calculated with Greenhouse–Geisser’s correction due to failure to meet the sphericity condition.

At baseline, no significant between-group differences were observed in any of the functional measures. Both groups demonstrated significant improvements in the BMC Total (total score for Bilateral Motor Coordination test), BMC Upper limbs (score for BMC sub-test for upper limbs) and SPr Total (total score for Sequencing Praxis test). In these tests within the Research group, scores increased significantly immediately following the intervention and remained elevated at follow-up, with a slight additional improvement observed after the break. In contrast, the control group exhibited a gradual, non-significant increase across consecutive measurement points, which culminated in a statistically significant difference after the final assessment.

Research group demonstrated significant improvement in the BMC Lower limbs (score for BMC sub-test for lower limbs), SPr Hands (score for SPr sub-test for hands), SPr Fingers (score for SPr sub-test for fingers) and PrVC Time (time of the execution of tasks in Praxis on Verbal Command test) while the Control group demonstrated significant improvement in the FG Time (time of the execution of tasks in Figure-Ground perception test). While the change in SPr Hands in the Research group maintains a similar dynamic to the previously discussed indicators (it increases immediately after the intervention and is maintained after follow-up), the changes in SPr Fingers and BMC Lower limbs resemble the trajectory of changes in the previously discussed results in the Control group (a significant change appears only after follow-up). Task performance time (in the Research group – PrVC task; in the Control group – FG task) also did not decrease significantly immediately after the intervention but improved significantly after follow-up.

The analysis of correlations between variables (without division into groups) is presented in Table 4, however, the results of those sub-tests that differed only in the side of the body (right–left) were omitted.

**Table 4:** Results of the correlation analysis between variables in whole group of participants.

	PPr	CPr	PrVC	PrVC Time	SPr Hands	SPr Fing.	SPr Total	KIN	FG	FG Time	MFP	MFP Time	LTS	GMC	BMC UL	BMC LL	BMC Total
PPr		0.14	**0**.**59**	**−0**.**33**	**0**.**48**	**0**.**61**	**0**.**56**	**−0**.**33**	**0**.**28**	−0.10	**0**.**42**	−0.20	−0.23	−0.01	**0**.**38**	**0**.**48**	**0**.**41**
CPr	0.14		0.15	−0.09	0.08	0.09	0.08	−0.04	0.16	−0.03	0.05	0.08	−0.21	−0.14	0.16	0.10	0.14
PrVC	**0**.**59**	0.15		**−0**.**53**	**0** . **75**	**0**.**62**	**0** . **74**	**−0**.**34**	**0**.**39**	0.13	**0**.**55**	0.07	**−0**.**33**	−0.14	**0**.**54**	**0**.**62**	**0**.**58**
PrVC Time	**−0**.**33**	−0.09	**−0**.**53**		**−0**.**35**	−0.19	**−0**.**30**	0.07	0.01	0.15	**−0**.**26**	0.02	0.09	**0**.**32**	**−0**.**41**	**−0**.**32**	**−0**.**40**
SPr Hands	**0**.**48**	0.08	**0** . **75**	**−0**.**35**		**0** . **76**	**0** . **96**	**−0**.**40**	**0**.**45**	0.22	**0**.**49**	0.15	−0.21	−0.01	**0**.**65**	**0**.**68**	**0**.**69**
SPr Fing.	**0**.**61**	0.09	**0**.**62**	−0.19	**0** . **76**		**0** . **91**	**−0**.**46**	**0**.**42**	0.24	**0**.**50**	0.02	**−0**.**25**	0.05	**0**.**59**	**0**.**63**	**0**.**63**
SPr Total	**0**.**56**	0.08	**0** . **74**	**−0**.**30**	**0** . **96**	**0** . **91**		**−0**.**45**	**0**.**48**	**0**.**26**	**0**.**52**	0.10	**−0**.**26**	0.03	**0**.**65**	**0**.**69**	**0**.**69**
KIN	**−0**.**33**	−0.04	**−0**.**34**	0.07	**−0**.**40**	**−0**.**46**	**−0**.**45**		−0.06	0.05	−0.02	0.01	0.12	−0.04	−0.21	**−0**.**26**	−0.23
FG	**0**.**28**	0.16	**0**.**39**	0.01	**0**.**45**	**0**.**42**	**0**.**48**	−0.06		**0**.**64**	**0**.**34**	0.04	−0.17	0.23	0.22	0.16	0.20
FG Time	−0.10	−0.03	0.13	0.15	0.22	0.24	**0**.**26**	0.05	**0**.**64**		0.19	0.18	−0.16	**0**.**32**	−0.09	−0.11	−0.11
MFP	**0**.**42**	0.05	**0**.**55**	**−0**.**26**	**0**.**49**	**0**.**50**	**0**.**52**	−0.02	**0**.**34**	0.19		−0.02	−0.18	0.10	**0**.**32**	**0**.**25**	**0**.**32**
MFP Time	−0.20	0.08	0.07	0.02	0.15	0.02	0.10	0.01	0.04	0.18	−0.02		−0.15	0.15	0.14	0.17	0.17
LTS	−0.23	−0.21	**−0**.**33**	0.09	−0.21	**−0**.**25**	**−0**.**26**	0.12	−0.17	−0.16	−0.18	−0.15		0.10	−0.22	−0.22	−0.22
GMC	−0.01	−0.14	−0.14	**0**.**32**	−0.01	0.05	0.03	−0.04	0.23	**0**.**32**	0.10	0.15	0.10		-0.21	**-0**.**26**	-0.22
BMC UL	**0**.**38**	0.16	**0**.**54**	**-0**.**41**	**0**.**65**	**0**.**59**	**0**.**65**	-0.21	0.22	-0.09	**0**.**32**	0.14	-0.22	-0.21		**0** . **78**	**0** . **98**
BMC LL	**0**.**48**	0.10	**0**.**62**	**-0**.**32**	**0**.**68**	**0**.**63**	**0**.**69**	**-0**.**26**	0.16	-0.11	**0**.**25**	0.17	-0.22	**-0**.**26**	**0** . **78**		**0** . **87**
BMC Total	**0**.**41**	0.14	**0**.**58**	**-0**.**40**	**0**.**69**	**0**.**63**	**0**.**69**	-0.23	0.20	-0.11	**0**.**32**	0.17	-0.22	-0.22	**0** . **98**	**0** . **87**	

PPr – Postural Praxis; CPr – Constructional Praxis; PrVC – Praxis on Verbal Command; SPr – Sequencing Praxis; KIN – Kinesthesia; FG – Figure-Ground perception; MFP – Manual Form Perception; LTS – Localization of Tactile Stimuli; GMC – Gross Motor Coordination test; BMC – Bilateral Motor Coordination test; UL – Upper limbs; LL – Lower limbs. The table presents rs values after Spearman's rank correlation analysis. Statistically significant values are bold. High correlations according to Guilford's interpretation (*r*s > 0.5) are marked in red, while very high correlations according to Guilford's interpretation (*r*s > 0.7) are underlined. Shaded are correlation values that should not be analysed because some values are included in others (SPr Hands and SPr Fing are included in SPr Total; BMC UL and BMC LL are included in BMC Total).

**Figure 1:**
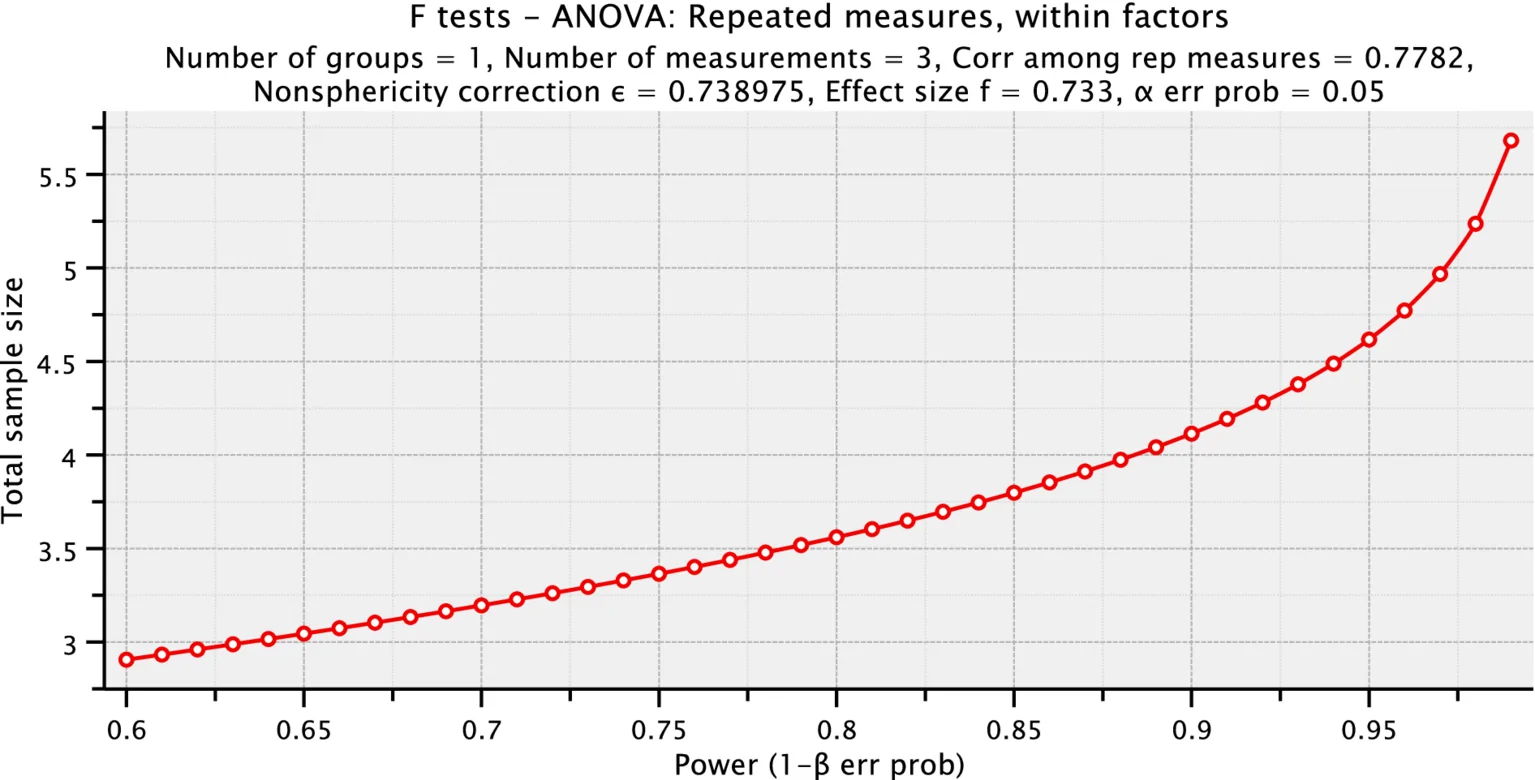
*Post-hoc* power analysis for BMC Total in Control group.

**Figure 2:**
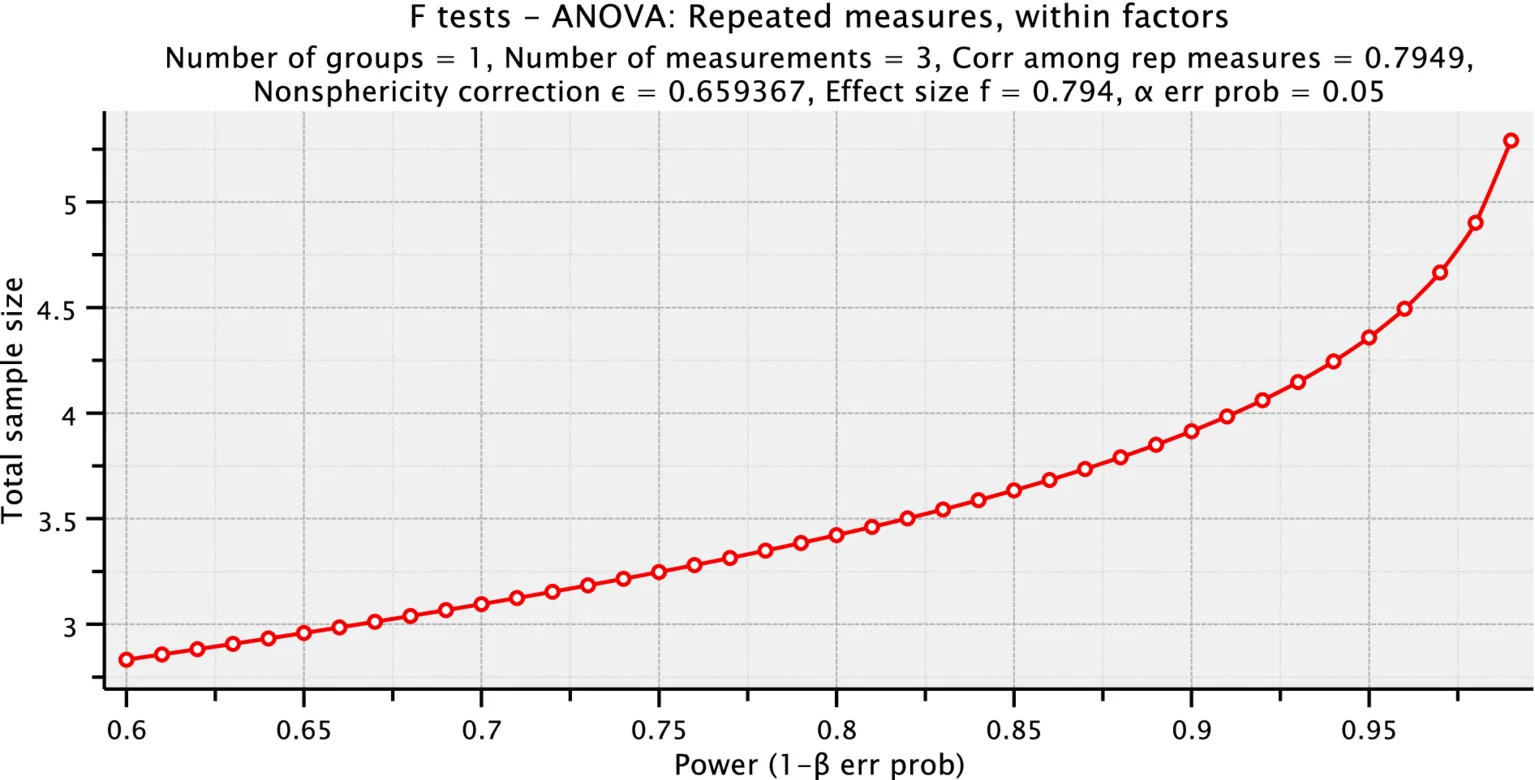
*Post-hoc* power analysis for BMC Total in Research group.

**Figure 3:**
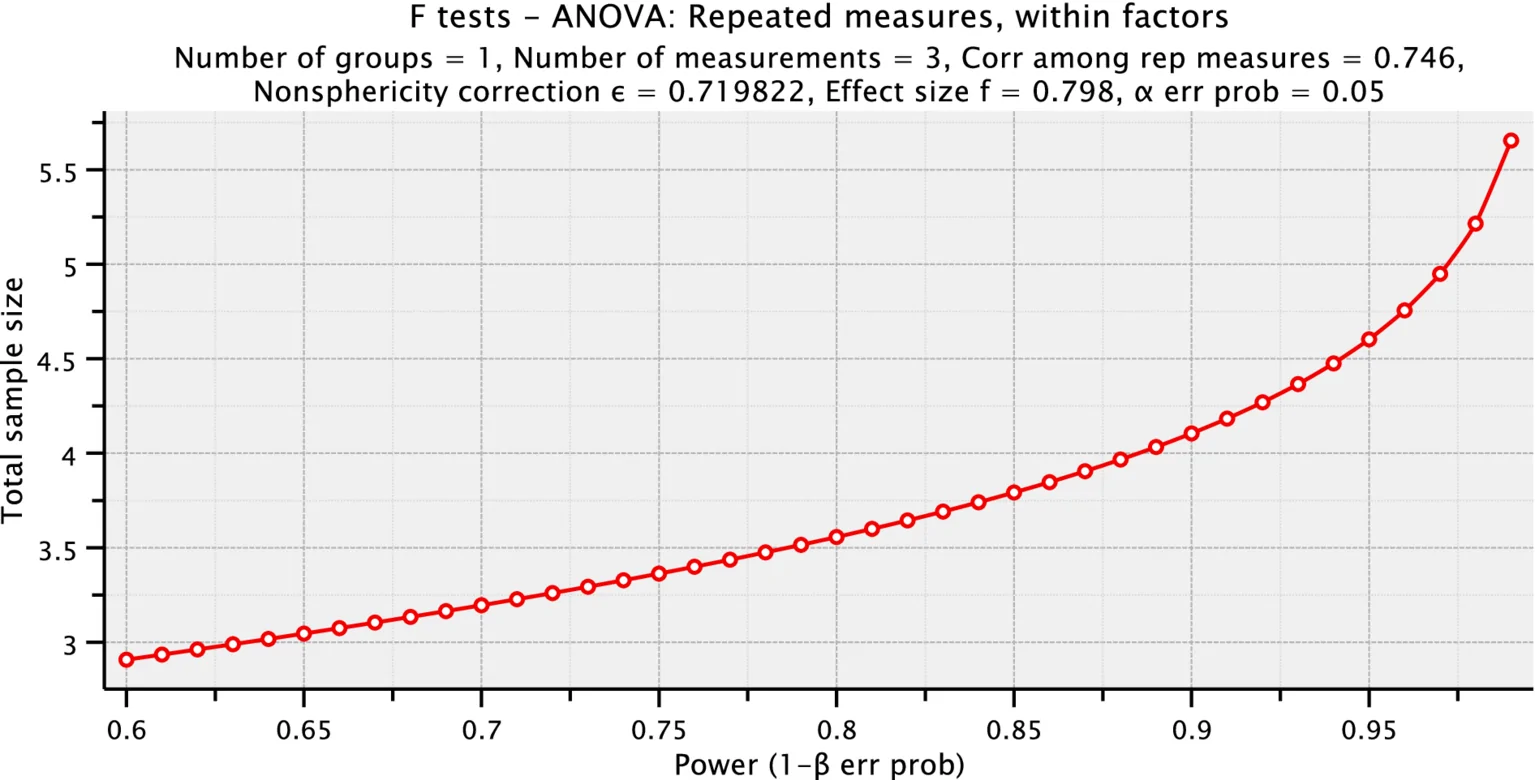
*Post-hoc* power analysis for BMC upper limbs in Control group.

**Figure 4:**
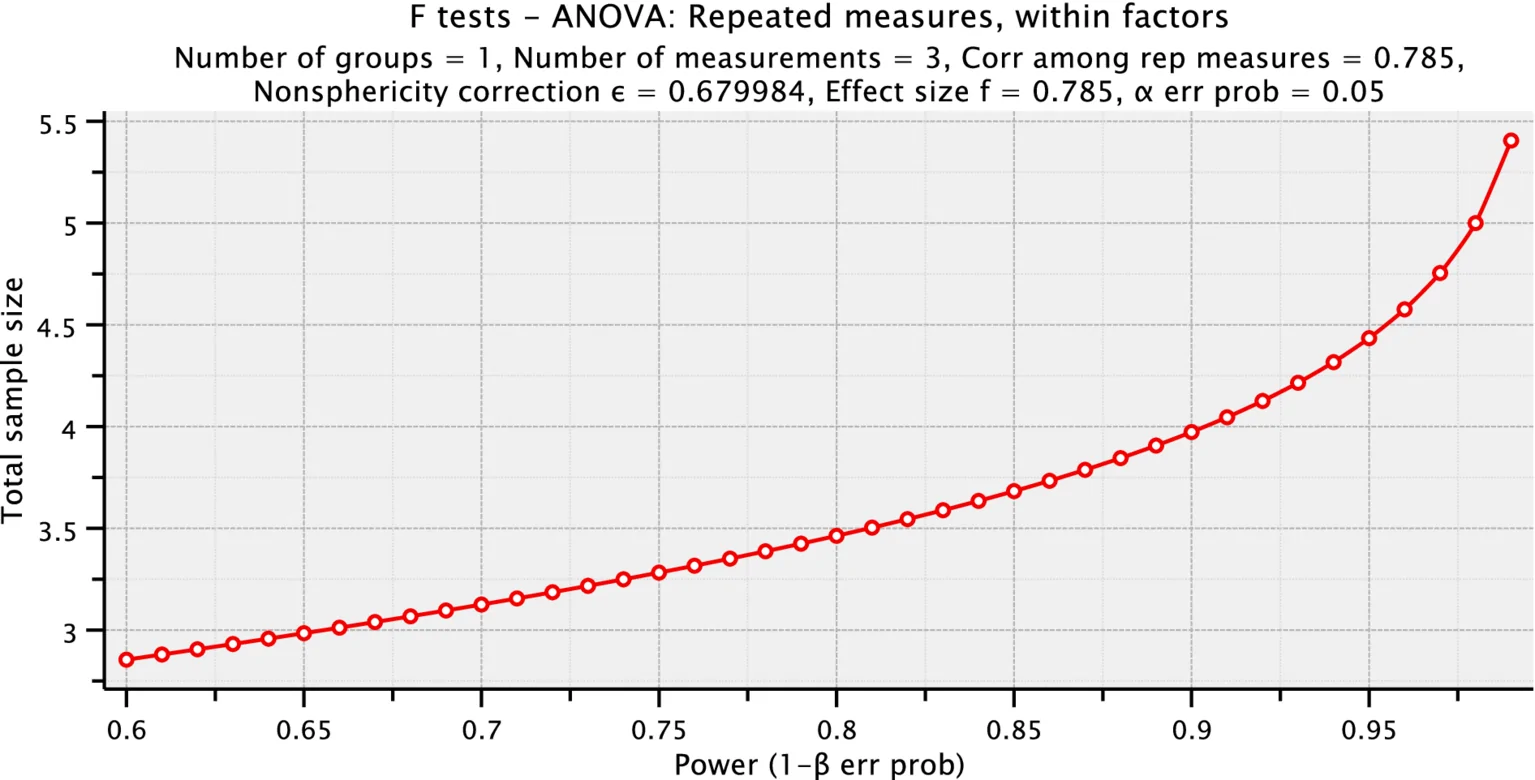
*Post-hoc* power analysis for BMC upper limbs in research group.

**Figure 5:**
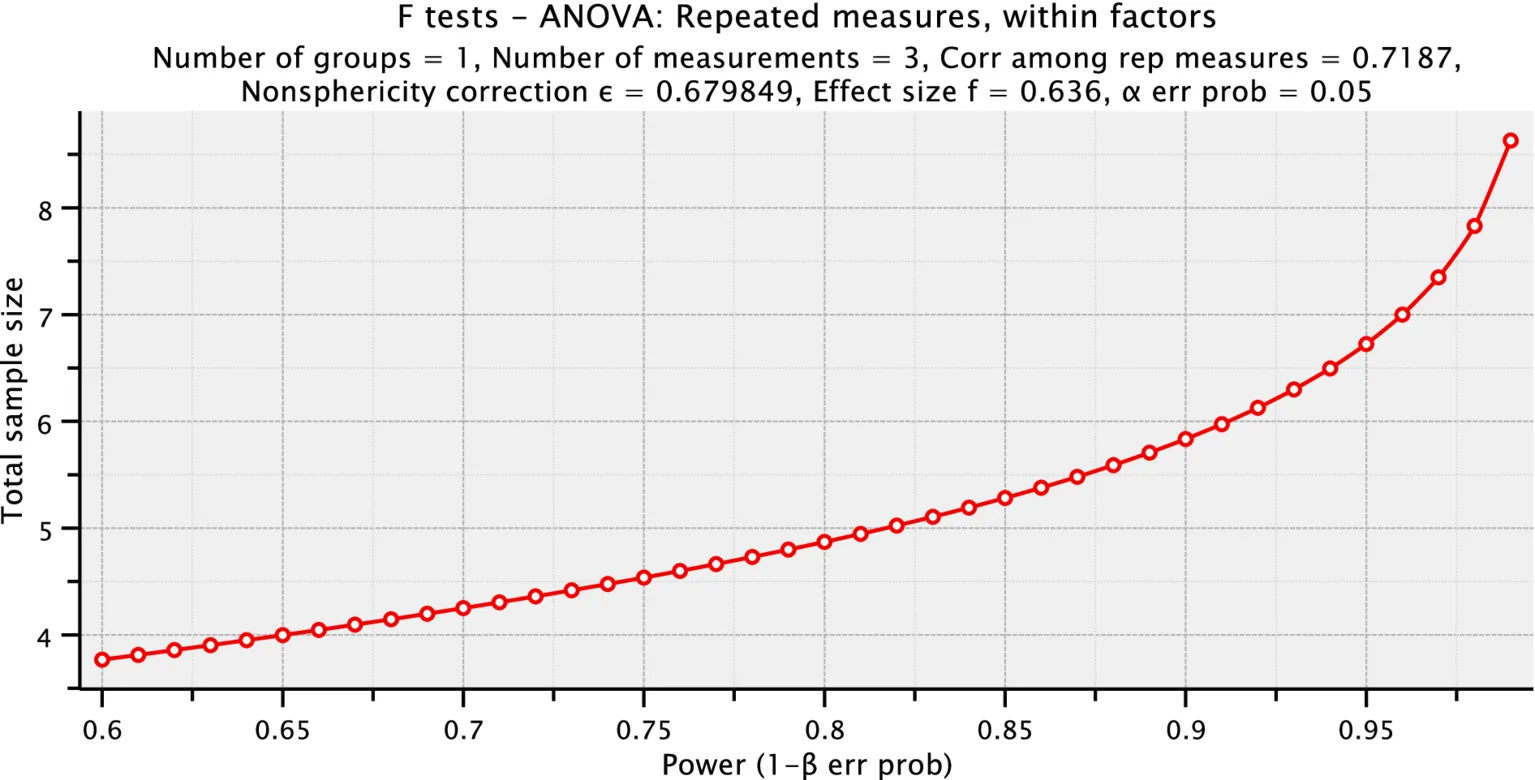
*Post-hoc* power analysis for BMC lower limbs in research group.

**Figure 6:**
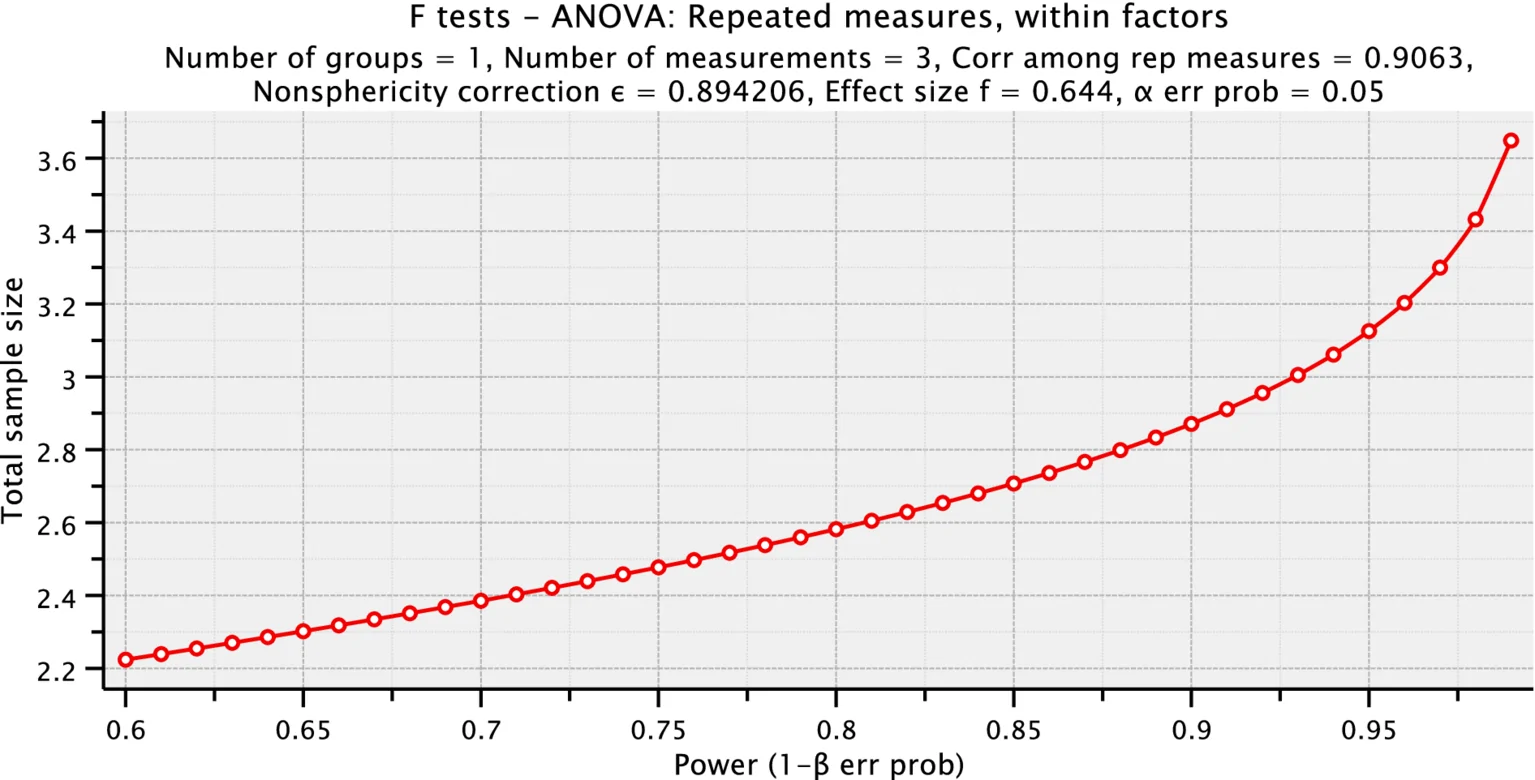
*Post-hoc* power analysis for SPr Total in control group.

**Figure 7:**
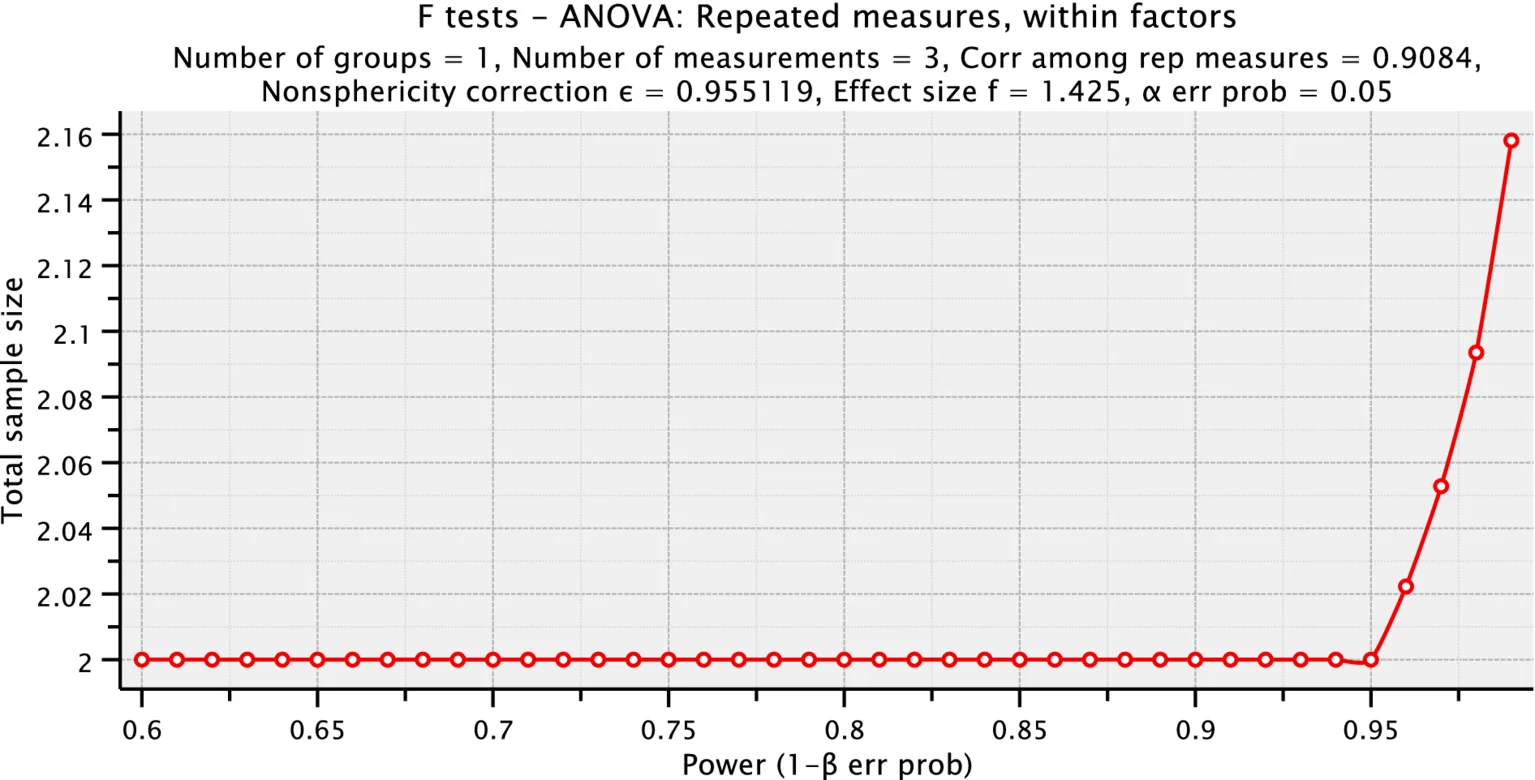
*Post-hoc* power analysis for SPr Total in Research group.

**Figure 8:**
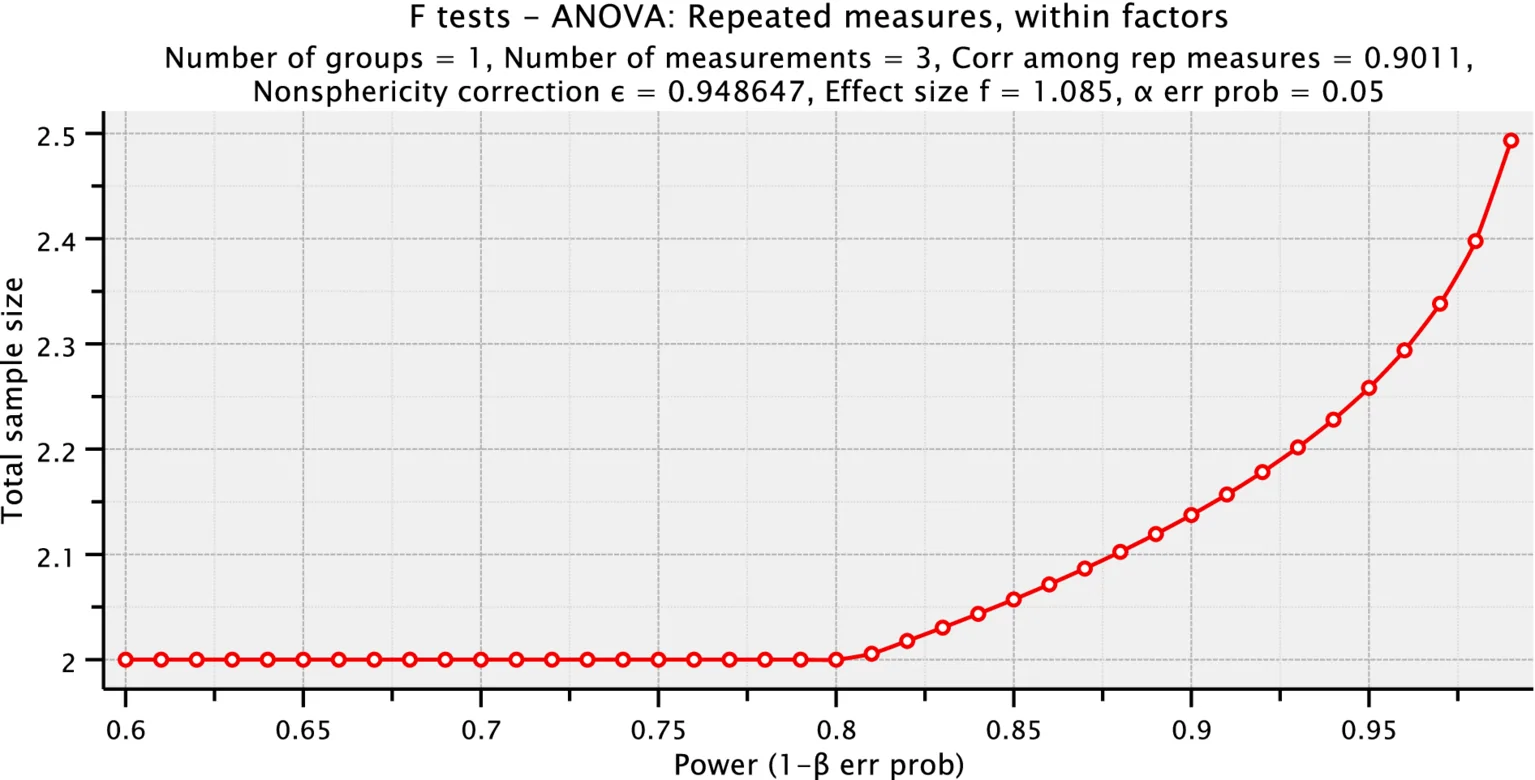
*Post-hoc* power analysis for SPr hands in research group.

**Figure 9:**
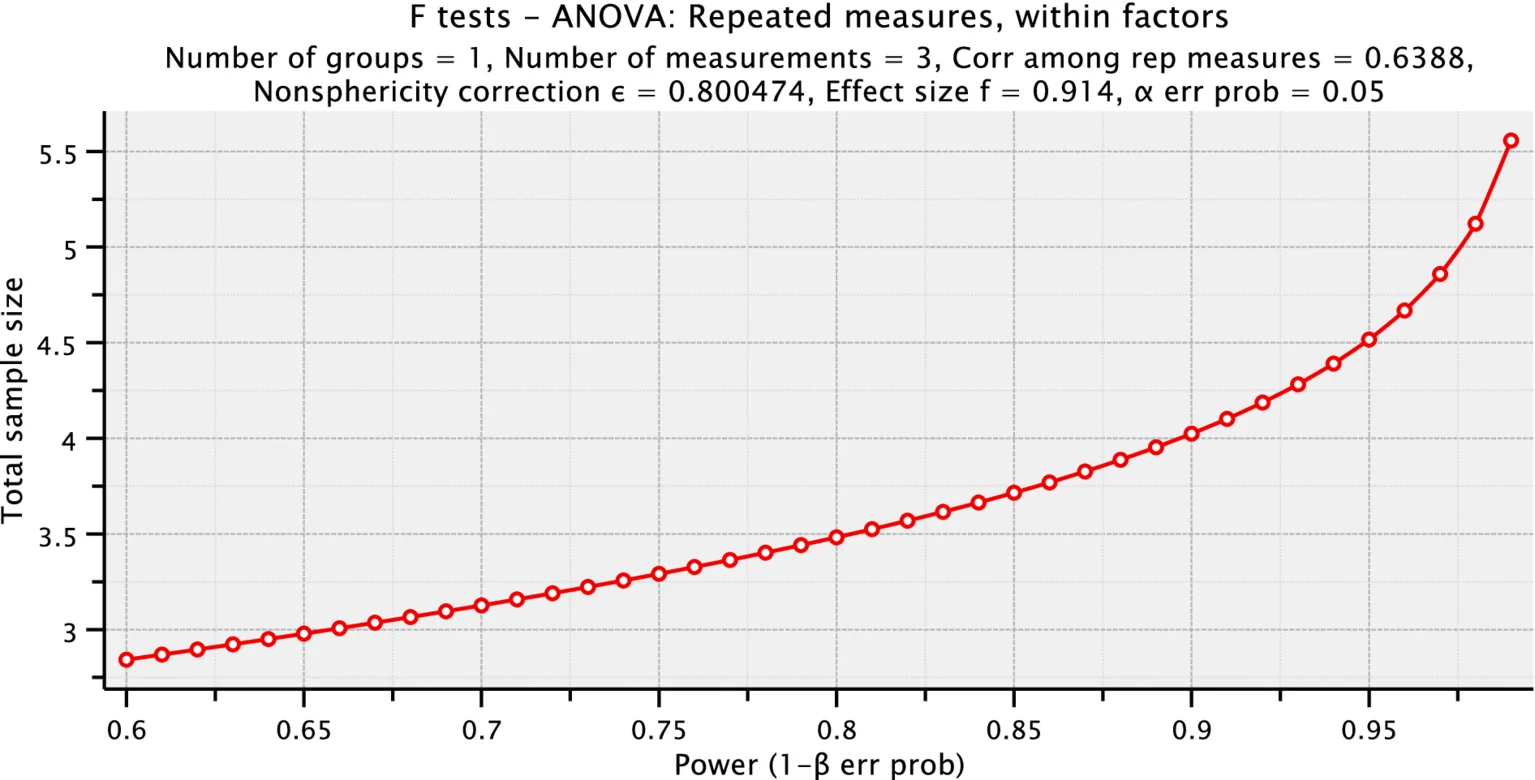
*Post-hoc* power analysis for SPr Fingers in research group.

**Figure 10:**
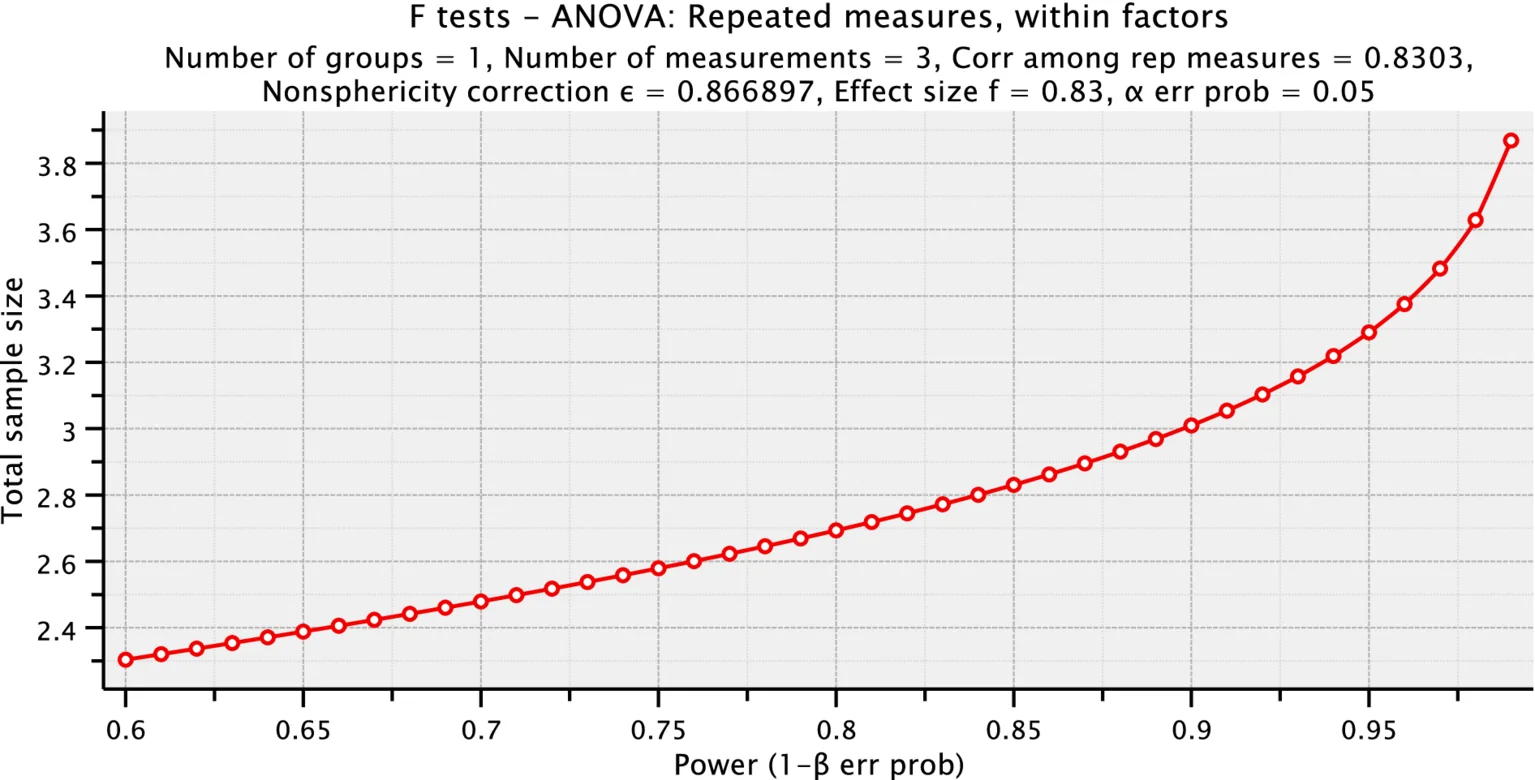
*Post-hoc* power analysis for PrVC time in research group.

**Figure 11:**
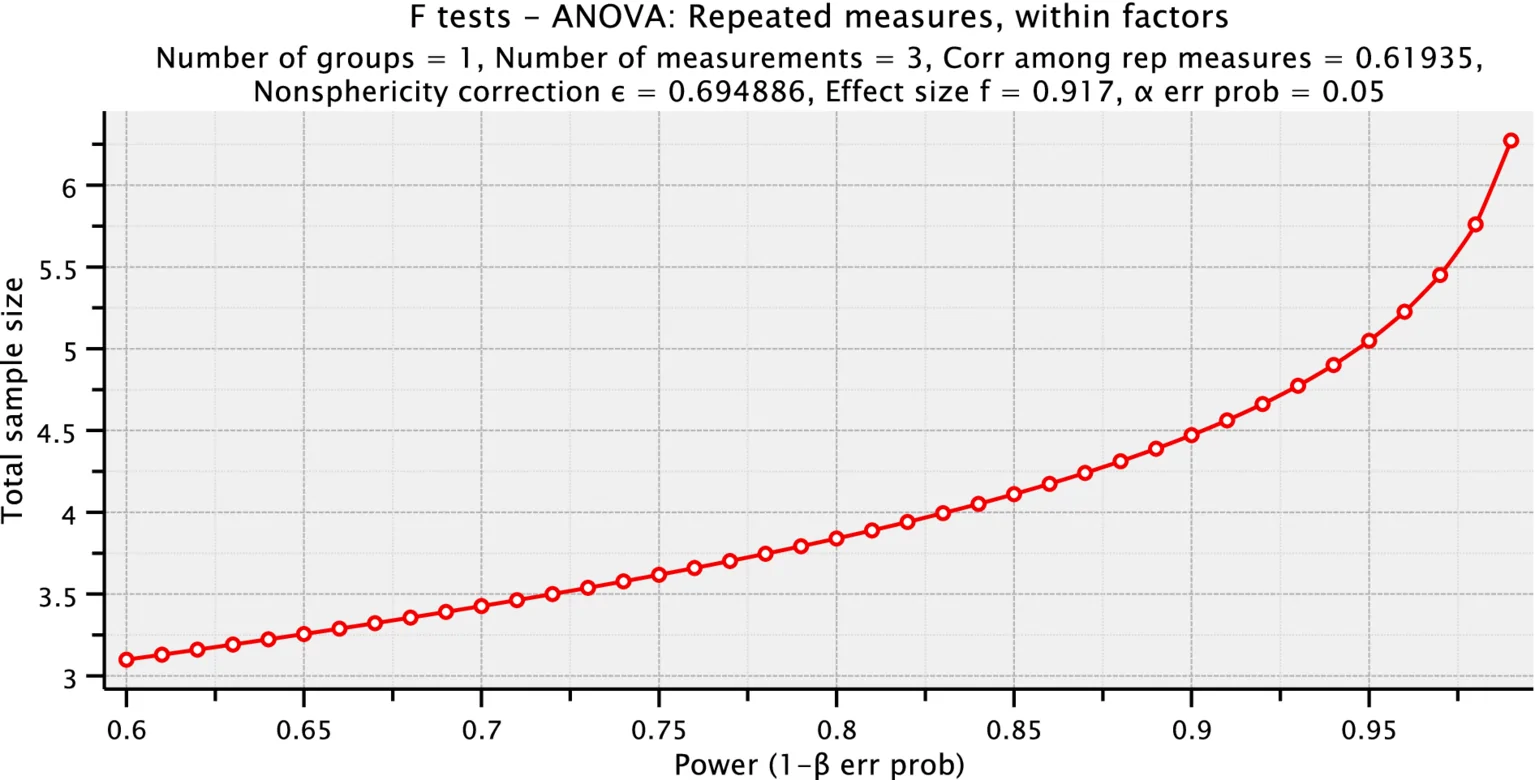
*Post-hoc* power analysis for FG Time in Control group.

To further verify the effect of the Bilateral Integration intervention on the significant outcomes, a *post hoc* power analysis was performed using G*Power (version 3.1.9.7). The analysis was conducted under the “ANOVA: Repeated measures, within factors” framework, incorporating the following parameters: effect size (f), α error probability, total sample size, number of groups, number of measurement points, correlation among repeated measures, and the nonsphericity correction coefficient (ε), calculated according to the Greenhouse–Geisser adjustment. Detailed analyses are presented in Figures 1–11. The *f* value needed for analysis in the G*Power program was obtained from the transformations of η^2^ in the case of parametric ANOVA and Kendall’s W in the case of non-parametric ANOVA.

## Discussion

The conducted research and the analysis of its results allow us to determine whether the type of physical exercise performed influences cognitive functioning in a specific way. The Preliminary Report article demonstrated significant changes in both groups in the BMC and SPr tests, indicating a different trajectory of change (both groups experienced significant improvement, but in the Research group the change occurred faster, as it occurred after the intervention had ended) (17). This paper allows for a more advanced assessment, as it also includes sub-tests.

### Improvement in SPr

In both analyzed groups, significant improvements in the overall SPr test score were observed, indicating that both exercises with and without midline crossing were associated with beneficial changes in the studied functions. The final results were similar in both groups. The differences concerned the timing of significant improvement. In the Research group, the change was visible already in the second measurement (t_1_) and persisted in the subsequent one, whereas in the Control group, significant improvement was noted only after follow-up (t_2_). This may indicate a different rate of therapeutic effect, with comparable final results. At the same time, no significant differences were found between the groups at individual time points. However, it is worth noting that despite the lack of statistical significance in the baseline measurement, the mean values indicated some variation in the initial level, which could have influenced the magnitude of the observed changes in the intragroup analyses (the Research group started from a lower level, but the results ultimately equalized). The obtained results suggest that both interventions may be effective, but potential differences in the dynamics of improvement require further research using analyses that allow for direct comparison of the trajectories of change over time.

In the analysis of the subtest concerning sequencing praxis of the hands (SPr Hands), significant improvement over time was observed only in the Research group, with a large effect size (η^2^ = 0.541). In the Control group, a gradual increase in mean values was observed, but this change did not reach statistical significance. In the analysis of the subtest concerning sequencing praxis of the fingers (SPr Fingers), significant improvement over time was observed only in the Research group, with a large effect size (η^2^ = 0.455). In the Control group, a gradual increase in scores was observed, but this did not reach statistical significance. Despite the different trajectories of change in both subtests, the final scores of both groups were similar, suggesting that the differences were primarily related to the dynamics of improvement rather than the final effect of the intervention. These results indicate that both interventions can lead to similar outcomes, although the rate of change may differ.

The pattern of changes in the SPr test and subtests suggests that differences between groups are primarily related to the rate of change rather than the final effect of the intervention. The Research group, which started from a lower level in the subtests and the entire test, demonstrated greater improvement and achieved significance earlier or more clearly in the within-group analyses. The Control group improved gradually, allowing for a significant change in the overall score, but not in individual components (hands and fingers) - this change may occur naturally due to development, however, it is difficult to access because it has not been studied away from the intervention. In summary, the study results indicate that both forms of intervention were effective in improving sequencing praxis function. Differences in the timing of significant changes in the subtests may reflect different rates of functional adaptation, while the final therapeutic effect was comparable between the groups.

### Improvement in BMC

In both analyzed groups, significant improvements in the overall BMC test score were observed, indicating that both exercises with and without midline crossing were associated with beneficial changes in bilateral coordination. The final scores were similar in both groups, with differences in the timing of significant improvement. In the Control group, the change was visible in the last measurement (t_2_), while in the Research group, significant improvement was already visible in the second measurement (t_1_) and persisted at the subsequent time point. This may indicate a different rate of increase in the intervention effect, with a comparable final result. At the same time, no significant differences were found between the groups at individual time points. It is worth noting that despite the lack of statistical significance in the baseline measurement, the mean values indicated some variation in the initial level, which could have influenced the magnitude of the observed changes in the intragroup analyses.

In the analysis of the subtest concerning bilateral upper limb coordination (BMC Upper Limbs), significant improvement over time was noted in both groups. In the Control group, the change was gradual and significant at the later measurement point (t_2_ > t_0_ and t_2_ > t_1_), whereas in the Research group, improvement was already visible between t_0_ and t_1_ and was maintained at the next measurement (t_2_ > t_0_). Final values at t_2_ were similar, suggesting that the differences between the groups primarily relate to the rate of adaptation rather than the final effect of the intervention. In the subtest concerning bilateral coordination of the lower limbs (BMC Lower Limbs), significant improvement over time was observed only in the Research group, with a moderate effect size (η^2^ = 0.288). In the Control group, changes did not reach significance, although mean values gradually increased.

The pattern of changes in the BMC test and subtests indicates that the differences between the groups primarily relate to the rate of improvement rather than the final effect of the intervention. The Research group, which demonstrated early improvement in the lower limb subtest and rapid improvement in the upper limb subtest, may have been more sensitive to the exercise modality used, while the Control group improved gradually, achieving similar final scores for the hands and total scores. The BMC analysis results suggest that both forms of intervention are effective in improving bilateral coordination function of the upper limbs (although with different trajectories and dynamics of change), whereas the improvement in lower limb function depended on the type of exercise and occurred significantly only in Research group. The differences in the rate of change emphasize the importance of the type and degree of lower limb involvement in the training program, while maintaining a comparable final effect for the upper limbs and total scores.

### An attempt to explain the improvement in SPr and BMC

Improvement in SPr and BMC tests requires improved rhythm, counting, auditory-motor integration, somatognosis, movement control, inhibitory control, and sequence recall. Emphasizing this aspect is particularly important because, at the neural level, many of the aforementioned elements (or their components) are implemented with significant participation of the parietal cortex. It participates in spatial processing and movement initiation (26); movement planning (27); orientation in space and in one’s own body schema (28); movement observation, sensory-motor integration, and hand movement control (reaching, grasping, and pointing) (29), spatial awareness, and motor orientation and control (30). Considering the role of the parietal cortex in the implementation of the these functions (and thus in the abilities measured by the SPr and BMC tests) is important because research indicates that movements crossing the body’s midline additionally stimulate the parietal cortex (especially the SPC) (12). From the point of view of the results of this study, it is also important to emphasize the role of the parietal cortex in hand function (29), for example, in writing (31), as well as in mental representations of hand movements (32). Studies suggesting that movements crossing the body’s midline influence SPC stimulation (12) could therefore also explain the fact that significant changes in the SPr Hands and even SPr Fingers subtests were observed in the Research group. It should also be emphasized that planning, executive control, working memory, and the organization of action sequences are also crucial in SPr and BMC tasks, in which the DLPFC plays a significant role (33). These tasks require rhythm, ongoing control over alternation, and maintaining a motor pattern, which engages, among other things, prefrontal mechanisms responsible for monitoring and modulating motor activity. The previously mentioned electroencephalographic studies also confirm DLPFC activation during midline crossing movements (12). Differences in the dynamics of changes between the Control and Research groups may, in turn, result from the fact that midline crossing movements are associated with the activation of plasticity-related potentials (13). Therefore, functional changes mediated by the DLPFC and SPC may occur more rapidly due to the nature of neuroplasticity. However, many studies point out that the PPC (posterior parietal cortex) is also involved in the representation and generation of hand movements (34–36). Therefore neuroimaging studies would be necessary to confirm whether movements crossing the midline affect the PPC similarly to the SPC and DLPFC. Neuroimaging studies have shown that in sequence-based tasks (which are the nature of the SPr and BMC tests), frontoparietal networks are activated (37), which could again confirm that bilateral integration exercises crossing the midline activate prefrontal and parietal areas.

### Improvement in hand functions

It’s also worth noting that regardless of the group, the interventions improved fine motor skills, as evidenced by improved BMC parameters in the upper limbs and the overall SPr score (only the hands are used in this test). This is particularly interesting because the interventions were based solely on gross motor exercises. This demonstrates that Bilateral Integration Exercises can influence fine motor skills, even if they focus on gross motor skills. It’s worth emphasizing that not all gross motor interventions affect fine motor skills (38), and researchers emphasize that if improvement in fine motor skills is expected, it’s best to combine a gross motor intervention with fine motor exercises (39). However, the correlation between gross and fine motor skills is emphasized (38) and the interaction of various functional domains, such as the mutual influence of gross motor skills, fine motor skills, and language (40). The described changes in fine motor skills may also be based on a functional transfer mechanism, because both gross and fine motor skills – despite topographic differences (both in the body and in the cerebral cortex) – share common processual elements, such as reliance on postural control, sensorimotor integration, coordination skills, and movement planning (41, 42). Furthermore, improving proximal function (motor control) may have created conditions for improving distal function (fine motor skills), consistent with the proximo-distal direction of development (43). Therefore, the direct effect of gross motor skills training on fine motor skills does not always occur in short-term interventions, but this influence is explainable in terms of developmental mechanisms – improving global movement organization and bilateral coordination may have created more favorable conditions for the performance of precision tasks by improving overarching planning and stabilization mechanisms. Bilateral Integration Exercises may therefore constitute a more general developmental intervention, and its impact can be explained based on the promotion of fundamental movement skills (44), which may promote broader changes in motor functioning. Moreover, the fact that in this study both interventions influenced functions performed with the help of hands, but only the exercises involving crossing the midline additionally influenced functions related to precise movements of the fingers, as well as motor control and rhythm of movements of the lower limbs, suggests that in general developmental and holistic interventions it is worth considering those exercises of Bilateral Integration that include an element of crossing the midline of the body.

### Bilateral integration exercises in DCD

Improvements in bilateral coordination, movement sequencing, and control over movement alternations, however, may be explained not only by potential effects on frontoparietal areas, but also by activity in the corpus callosum, responsible for interhemispheric communication (45), or the cerebellum, responsible for precise movement timing, error correction, and motor sequence learning (46).

The improvements observed in the SPr and BMC tests in this study indicate that interventions based on Bilateral Integration Exercises may be very valuable in children with DCD symptoms. Previous literature indicates that difficulties crossing the midline are characteristic of children with DCD, and that midline crossing behaviors are associated with complex interhemispheric communication (47). In the context of bilateral coordination, children with DCD have been observed to have difficulties with both unimanual and bimanual tasks, indicating significant motor control deficits on both sides of the body (48). Therefore, motor training that supports bilateral coordination may be considered particularly effective in improving functional outcomes in children with symptoms of DCD.

Similarly, the literature on motor sequence learning has shown that motor training can lead to changes in the activity of areas involved in planning and executing sequential tasks, which is consistent with the observed improvements in the SPr and BMC. Studies on the involvement of the prefrontal cortex, including prefrontal areas such as the DLPFC, in motor sequence learning emphasize that processes related to the preparation and planning of subsequent movement elements are crucial for improving the precision and speed of sequence execution (49).

Studies in a population of children with DCD have shown that their reduced motor control was also reflected in reduced activity in the left SPC during movement correction (50). Other studies have noted reduced activation of the right DLPFC in children with DCD (51). Interventions that indirectly suggest an impact on the SPC and DLPFC may therefore be very valuable in planning therapeutic programs for children with DCD symptoms.

This study also supports other studies that provide evidence that motor interventions in children with DCD improve motor coordination, fine motor skills (52), hand-eye coordination (53), and cognitive function (54). Bilateral Integration Exercises may therefore be another intervention that may be very valuable in disorders similar to DCD.

### Improved time in PrVC and FG tests

An interesting, though unexpected, result was an improvement in the speed of performance on some tests – in the Control group, this was the Figure-Ground Perception (FG) test, while in the Research group, it was the Praxis on Verbal Command (PrVC) test. It should be noted, however, that only the speed of performance on these tasks improved significantly, not their accuracy (which did not show a significant change). The results of this study therefore demonstrate that motor interventions in the form of Bilateral Integration Exercises in children with DCD can lead to selective improvements in the speed of task performance, without significant changes in response accuracy. This pattern suggests that, for these tasks, the interventions primarily affect executive efficiency and processing time, rather than the level of motor or perceptual competence itself. The literature on DCD emphasizes that these children often achieve accurate accuracy in tasks but perform them more slowly, which is associated with difficulties in movement planning, sequence prediction, and sensory integration (51, 55, 56).

The effect in the Research Group may be explained by improved motor planning and interhemispheric integration. Tasks that cross the midline require bilateral coordination and cooperation between both hemispheres, as well as spatial organization of movement relative to the body axis. Neurocognitive models of DCD, such as the Internal Modeling Deficit Hypothesis (56, 57), posit that children with DCD have difficulty creating and updating internal models of movement, which increases the time it takes to prepare and execute actions. The improved performance speed in the praxis task may reflect more efficient use of these models and a reduction in the cost of motor preparation. In the Control group, the improvement in FG test performance time may be related to the Alternating Exercise, which is characterized by dynamic execution of movements in the sagittal plane. These exercises require rapid planning of motor sequences, attentional switching between motor paths, and bilateral coordination in a dynamic context (16). These mechanisms indirectly overlap with those important in the FG test, which requires stimulus selection, background isolation, context rejection, and rapid processing of visual information. The improvement in performance time in this task may therefore reflect the transfer of attention training and the efficiency of temporal–spatial processing from alternating movements to visual perception functions, consistent with motor-cognition transfer (58, 59). It is known that the efficiency of perceptual figure-background discrimination is influenced, among other things, by stimulus symmetry (60). It is therefore possible that symmetrical exercises influence the physical sense of symmetry, which translates into a more effective perception of symmetry in visual stimuli. However, this hypothesis would require further research.

The observed differences between the groups indicate that different intervention components target specific functional systems: midline-crossing components primarily influence the speed of movement execution in response to verbal commands, while dynamic alternating movements may improve perceptual processing and the speed of visual stimuli selection. These results are consistent with neurocognitive models of DCD, which emphasize the heterogeneity of children’s difficulties and the interplay between movement planning, attentional control, and task performance.

The obtained data suggest that task-motor interventions can reduce processing costs and improve executive processes, which manifests as shorter task completion times within a given functional system, without necessarily improving accuracy itself. Different exercise components (midline-crossing vs. alternating movements) can selectively influence the pace of task performance depending on the type of sensory-motor and cognitive load, which confirms empirical observations regarding the transfer of motor functions to perceptual functions in DCD. However, these are single parameters, so this aspect requires further research, e.g., measuring time in other perceptual tasks and praxis tasks, and, above all, measuring time separately for individual task stages: ideation, planning, and execution in the context of praxis or detection, interpretation, and reaction in the context of perception.

### Correlations

The correlation analysis (Table 4) included the results of all participants, without group division, to examine the structure of relationships between variables regardless of group membership. Correlation analysis often focuses primarily on the interdependence of variables rather than on intergroup differences. Many of the calculated correlation coefficients reached statistical significance. The CPr index was not significantly correlated with any other index, which could suggest its relative independence from the other examined indices. Therefore, constructional praxis may engage other mechanisms (e.g., visuospatial) rather than bilateral coordination or sensory perception. However, the literature indicates that dysfunctions in constructional praxis may be related to both visuospatial, perceptual, and motor aspects (61). The MFP Time indicator was also not significantly correlated with any of the other indicators, which may suggest that the speed of tactile shape perception performance is also a variable unrelated to the others studied. Among the indicators that achieved statistically significant correlations, were KIN, LTS, and GMC.

**Figure 12:**
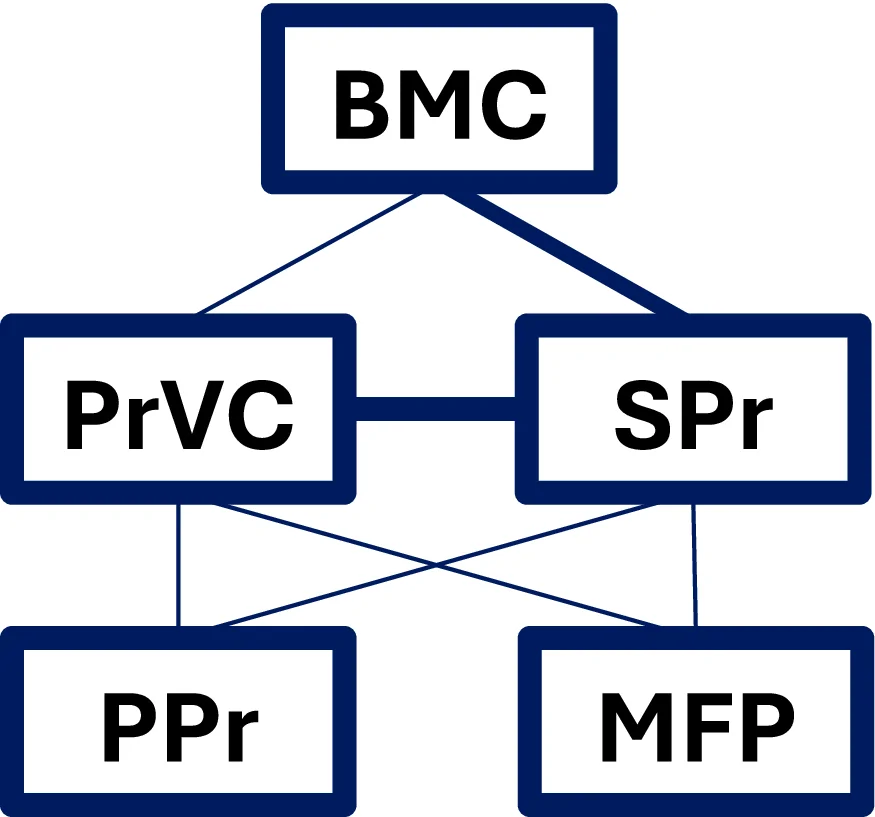
Correlation diagram between variables: PrVC, SPr, BMC, PPr, and MFP legend: The lines connecting the variables represent a correlation between them with *rs* >0.5; the medium-thick line (BMC-SPr) represents a correlation with *r*_s_ > 0.6; and the thickest line (PrVC-SPr) represents a very high correlation (*r*_s_ > 0.7). BMC – Bilateral Motor Coordination; PrVC – Praxis on Verbal Command; SPr – Sequencing Praxis; PPr – Postural Praxis; MFP – Manual Form Perception.

The cutoff point for further interpretation was set at Guilford’s high correlation threshold of |*r*| > 0.5 (62). However, correlation coefficients that result from being components of each other will be omitted (SPr Total is the sum of SPr Hands and SPr Fingers; BMC Total is the sum of the BMC Upper Limbs and BMC Lower Limbs subtests). After taking these assumptions into account, a clear, strongly integrated system of relationships between the variables: PrVC, SPr, BMC, PPr, and MFP emerges – to clearly present it, a diagram was developed (for better clarity, it does not include subtests) (Figure 12). This is a system in which PrVC and SPr seem to be the central variables, because they are related to all the others in the cluster.

The presented pattern of correlations may suggest that executing movements on verbal commands is not an isolated function but reflects a complex process integrating movement representation, sequential organization, and bilateral control.

High correlations between PrVC and SPr (especially SPr Hands) indicate that the ability to execute movements on verbal commands is closely related to the ability to organize and reflect movement in an orderly sequence. At the same time, strong correlations with BMC (especially BMC Lower Limbs) suggest that efficient execution of movements on verbal commands requires effective cooperation between both sides of the body, which may reflect the involvement of interhemispheric integration mechanisms. It is also significant that PPr reaches a threshold of high correlation with PrVC and SPr (especially SPr Fingers), which may indicate the importance of perceptual-motor representations in movement planning. The relationship between PrVC, SPr and MFP may suggest that movement planning and reflection are somehow linked to tactile perception – other studies have shown that during active touch, movement and somatosensory signals are strongly integrated, and interference between them influences the perception of space and movement (63).

It is also worth considering the correlations of the “Time” indices (PrVC Time, FG Time, and MFP Time). It has already been noted that MFP Time did not demonstrate any statistically significant correlation, but this is not the case for the other indices. Not surprisingly, the only high correlations of these indices are when compared with the test results from which they were derived – a high correlation was found between FG and FG Time (rs = 0.64) and between PrVC and PrVC Time (rs = −0.53). However, the direction of the relationship is interesting, as the correlation coefficients suggest that the longer the FG task execution time, the greater its accuracy, while a shorter PrVC task execution time correlates with better performance. It is therefore possible that more effective performance of perceptual tasks requires more time, which could be consistent with the so-called Fitts’ Law regarding speed-accuracy trade-off (64). More effective performance of motor tasks, on the other hand, was associated with faster performance, which may indicate the automation of movement planning processes – it can be assumed that as the integration of the praxis system increases, the demand for cognitive control decreases, which translates into shorter task execution times. Other studies have shown that motor learning is accompanied by optimization of internal models, resulting in more economical and faster movement execution and less load on control structures (65).

Overall, the results suggest the existence of a relatively homogeneous, higher-level organization of praxis, encompassing planning, sequencing, and bilateral movement control. This pattern of relationships can be interpreted in light of neuropsychological models of praxis, which emphasize the role of frontoparietal networks in the planning and execution of purposeful movements. The strong connections between PrVC, SPr, and BMC suggest the involvement of an integrated system encompassing: parietal areas (creation and storage of movement representations and integration of visual-somatosensory information), premotor areas and DLPFC (planning, sequential organization, temporal control of action), primary and secondary motor cortex and subcortical structures (fluency and automaticity of movement execution), and the corpus callosum (interhemispheric communication).

From a neuropsychological perspective, the results suggest that praxis is not a function localized in a single brain region but results from the operation of a distributed, functionally integrated network, and bilateral integration is a prerequisite for the effective implementation of a motor program. Overall, the results support the concept of praxis as a multicomponent, hierarchically organized neurocognitive system, integrating movement representation, sequential organization, and bilateral coordination mechanisms within frontoparietal networks.

In the context of DCD, it is particularly significant that the PrVC and SPr occupy a central position in the dependency structure, demonstrating strong correlations with both the PPr and MFP, and above all, with the BMC. This confirms that the difficulties observed in children with DCD symptoms are not limited to reduced motor skills but encompass a more profound deficit in movement organization and planning (14, 51, 57).

The very high correlation between PrVC and SPr may indicate that one of the key mechanisms underlying motor difficulties may be a disturbance in the temporal organization of action. Children with DCD often demonstrate difficulty seamlessly combining movement elements into a coherent whole, which may reflect a deficit in the mechanisms responsible for programming motor sequences—both to verbal commands and during recall (14, 51, 57). The high correlations between PrVC, SPr, and BMC suggest that limited interhemispheric integration may also be a significant factor, affecting the synchronization of movements between both sides of the body, which may be reflected in both planning and recalling movement sequences.

Overall, the results indirectly support the model of DCD as a network disorder, encompassing dysfunctional integration between movement representation (parietal component), its sequential organization (frontal component), and mechanisms of automaticity (subcortical structures) and bilateral coordination (interhemispheric connections). This picture goes beyond the traditional understanding of DCD as “motor clumsiness” and indicates deeper disorders of the organization of goal-oriented actions, which is clearly confirmed in the literature (14, 51, 57).

### *Post-hoc* power analysis

It is worth emphasizing, however, that the key results were characterized by very high statistical power, indicating that the obtained effects were so pronounced that similar results could have been achieved even with a smaller number of participants (Figures 1–11). Although the sample size was smaller than in some studies of interventions based on bilateral integration or conducted among children with DCD or with symptoms of DCD, the conducted power analysis confirms that the sample size was sufficient to reliably verify the hypotheses.

### Limitations

One of the main limitations is that the study did not include a neuroimaging component. The previously discussed relationships are based on established neuropsychological knowledge (or constitute hypotheses that could be based on it), but the study does not provide direct evidence that Bilateral Integration Exercises activate specific neural centers. Therefore, it would be worthwhile to design a similar study supplemented with electroencephalography, functional magnetic resonance imaging, or functional near-infrared spectroscopy.

Another important limitation of the study is that no changes in perception were demonstrated (apart from a change in the time required to complete a visual perception task, which, however, is not a direct indicator of perception, but rather of its efficiency). This lack of evidence could be due to the lack of association between Bilateral Integration Exercises and cognition, but previous research suggests otherwise (11). It is highly likely, however, that the intervention was conducted too briefly. A 26-week study would likely be necessary, as many studies examining changes in cognitive functioning are conducted over 26 weeks (66–68) – this practice also appears in studies involving children (11). A longer intervention could therefore produce broader results.

Another limitation of the study was that linear regression could not be performed after the correlation analysis because the sample size did not meet the 50 + 8 k requirement. Although the sample size was sufficient to verify the research hypotheses, it was too small to conduct a reliable regression analysis.

A ‘Group × Time’ interaction analysis was also not conducted, so it is impossible to clearly determine which intervention is superior. However, the analyses clearly indicate the time points at which significant change occurred, as well as the tests or subtests in which change occurred only in one group. Constructive conclusions can be drawn from these analyses, but a ‘Group × Time’ interaction analysis would certainly provide even clearer statistical evidence.

The final major limitation of the study was the high dropout rate (31.25%), although this issue was discussed in detail in the Preliminary Report (17).

It should also be emphasized that a single, structured exercise intervention within a research study is not enough. It is important that exercise (especially in children with DCD symptoms) be a consistent part of the day and performed in a variety of contexts, as emphasized by numerous authors (69–71).

## Conclusion

The results of this study clearly indicate that exercises according to the Bilateral Integration program positively impact sequencing praxis, bilateral motor coordination, and the time to complete certain cognitive/executive tasks. In turn, the type of exercises performed (specifically, whether the exercises include an element of crossing the body’s midline) influences the trajectory of improvement in certain functions or the nature of the task in which performance speed improves.

Furthermore, correlation analysis showed that the results suggest the existence of a relatively uniform, higher level of praxis organization, related not only to ideation, planning, and execution of the motor act, but also to the coordination of both sides of the body, working memory, attentional concentration, auditory processing, auditory-motor coordination, and tactile-epicritic perception. The results clearly indicate the interdependence of bilateral motor coordination, sequencing praxis, praxis on verbal command, postural praxis, and manual form perception.

Overall, the results may suggest that it’s difficult to find an intervention that will impact a single extracted function in isolation, as there’s a clear network of interdependencies in psychomotor development. The observed improvements in sequencing praxis, bilateral motor coordination, and time of perform some tasks are also consistent with the proposed involvement of the DLPFC and SPC, which play key roles in executive control, motor planning, spatial integration, and the coordination of complex goal-directed movements. Although the present study did not directly assess neural activity, the findings support the rationale for considering Bilateral Integration exercises as an intervention engaging functions associated with these cortical networks.

However, the results clearly demonstrate that Bilateral Integration contributes to functional improvement in children with DCD symptoms, and the nature of the exercises selected may influence the trajectory of that improvement. Future studies incorporating neurophysiological or neuroimaging measures are warranted to directly examine the involvement of DLPFC and SPC in the observed functional changes.

## Data Availability

The datasets presented in this article are not readily available because this paper is a report of research for doctoral thesis. The full data cannot yet be publicly available until they are subjected to further detailed analysis as part of a doctoral thesis (which should be finalized by 2028). Use of these data in publications by other authors could pose a significant obstacle to the implementation of the doctoral thesis, which assumes innovative results. The corresponding author will later be able to share the data with interested researchers by e–mail contacting. Requests to access the datasets should be directed to bartosz.bagrowski@gpsk.pl.
